# Beam-Hopping Scheduling for High-Priority and Time-Sensitive Services in Low-Earth-Orbit Satellite Networks

**DOI:** 10.3390/s26196081

**Published:** 2026-09-25

**Authors:** Liang Gou, Yulei Nie, Wei Sun, Dapeng Qi, Ziwei Liu, Gengxin Zhang

**Affiliations:** 1 School of Communications and Information Engineering, Nanjing University of Posts and Telecommunications, Nanjing 210003, China; gouliang@njupt.edu.cn (L.G.); lzw@njupt.edu.cn (Z.L.); gengxin_zhang@126.com (G.Z.); 2 College of Integrated Circuit Science and Engineering, Nanjing University of Posts and Telecommunications, Nanjing 210023, China; 3 Unit 31150 of PLA, Nanjing 210007, China; sunwei_lijuan@163.com; 4 Unit 96946 of PLA, Beijing 100045, China; dapeng_qi@163.com

**Keywords:** beam-hopping scheduling, low-Earth-orbit (LEO) satellite networks, high-priority services, time-sensitive services, system gain maximization, heuristic algorithm, graph reinforcement learning

## Abstract

Beam-Hopping (BH) dynamically adjusts beam pointing and dwell time to overcome the rigid resource allocation of traditional fixed beam coverage. This paper addresses the BH scheduling problem in low-Earth-orbit (LEO) satellite networks for high-priority and time-sensitive services. A system-gain maximization model and a heuristic beam scheduling algorithm (HBSA) that pre-schedules critical traffic and greedily allocates remaining resources under interference and fairness constraints are proposed. Furthermore, this paper extends the framework with a graph reinforcement learning beam-hopping scheduling scheme (GRL-BHS) that captures structural network dependencies for near-optimal adaptive scheduling. Simulations against seven benchmarks show that the HBSA achieves near-optimal gain (within 2% of MWC and GRL-BHS), ensures high-priority satisfaction, maintains a Jain fairness index above 0.84 under heavy load, and reduces signaling overhead by an order of magnitude, while preserving a low polynomial complexity of $O(T\times J\times K+T\times K^{2})$. GRL-BHS offers the highest gain at the cost of offline training, whereas HBSA is the most balanced and practically deployable solution, providing a solid foundation for future intelligent BH scheduling in mega constellations.

## 1. Introduction

In recent years, low-cost rocket launch and advanced manufacturing technology have paved the way for the deployment of satellite Internet, especially LEO-based satellites. The low orbit altitude of LEO satellites makes the space–ground transmission delay very short. It is more suitable for delay-sensitive services and application scenarios, such as multimedia entertainment, Internet of Vehicles (IoV) and real-time control [1]. LEO satellites also achieve terminal miniaturization due to the low propagation loss. At the same time, dense constellation deployment ensures seamless and global coverage. Therefore, mega LEO satellite constellation networks have been highly valued by some governments and global industry and deployed extensively in recent years [2].

In satellite communication systems, the traditional fixed-beam coverage mode has the problem of rigid resource allocation, which cannot flexibly adapt to the dynamic changes in business requirements in time and space. Beam-hopping (BH) technology, as a key technology for next-generation high-throughput satellites and low-orbit networks, solves this problem by dynamically adjusting beam pointing and dwell time [3]. It has the advantages of dynamically matching business needs, improving resource utilization, reducing system costs, and being compatible with future communication technologies. The primary advantages of beam-hopping technology are as follows,

- Dynamically matching business requirements. There are significant differences in communication requirements in different regions at different times. BH technology can concentrate beams in high-demand areas by perceiving real-time needs, avoiding resource waste in low-activity areas. In addition, it can quickly adjust beam allocation to cope with local traffic surges caused by temporary events (such as competitions and disasters), thereby improving system capacity.
- Improving resource utilization. Traditional fixed beams need to continuously cover all areas, resulting in scattered power. BH only activates target areas, concentrates transmission power to improve signal-to-noise ratio and spectrum efficiency, and shares frequency and power resources through time-division multiplexing, avoiding idleness caused by fixed allocation.
- Reducing system costs. Compared with multi-beam full-coverage systems, BH can replace a large number of fixed beams with a small number of reconfigurable beams using phased array antennas, reducing the hardware cost and weight of satellites. At the same time, dynamic power allocation reduces ineffective radiation and lowers energy consumption.
- Being compatible with future communication technologies. The high-speed movement of LEO satellites leads to frequent changes in coverage. BH can switch quickly to maintain continuous services and can match the slicing and dynamic requirements of ground networks, supporting space–ground integrated resource scheduling. In addition, the pseudo-random hopping mode of BH technology enhances anti-interference capability, which is particularly important in military communications. Although it introduces challenges such as scheduling complexity, with the optimization of intelligent algorithms, BH has become a core technology driving satellite communications towards high-efficiency, intelligent, and on-demand services, laying the foundation for space–ground integrated networks.

However, in the application of high-priority and time-sensitive services, BH technology faces challenges such as delay jitter, beam-switching synchronization, and increased mobility management complexity. And the challenges of beam-hopping technology in high-priority and time-sensitive services are as follows:

- Delay jitter and deterministic service challenges. The beam dwell time of LEO beam-hopping networks is only at the millisecond level. The discontinuous coverage caused by beam switching leads to intermittent interruptions in traffic transmission and increased delay jitter. Coupled with the inherent long propagation delay of satellite communications, it is very challenging to meet the ultra-low latency QoS requirements of some services.
- Beam-switching synchronization and signaling overhead. Frequent beam switching requires strict synchronization between terminals and satellites. Signaling interactions (such as beam-switching commands and channel state feedback) occupy the already limited bandwidth, thereby reducing the efficiency of effective data transmission, especially for small-packet services (such as IoT).
- Increased complexity of mobility management. High-speed moving users (such as fighter jets and high-speed rail) require more frequent beam switching, which may exceed the system switching capability, leading to an increase in link interruption probability and switching failure rate.

Existing beam-hopping scheduling algorithms mostly focus on business distribution and its dynamic changes, aiming to maximize business throughput while considering user fairness, inter-beam interference suppression, and link status, but rarely take user priority and low-latency requirements into account, which limits the application capability of time-sensitive services in LEO BH satellite communication networks [4]. Therefore, this paper proposes beam scheduling methods for high-priority and time-sensitive services in LEO satellite networks, which takes the satisfaction of high-priority and time-sensitive services as an important goal and optimizes the beam-hopping pattern while considering interference suppression, business distribution, and fairness principles.

The remainder of this paper is organized as follows. Section 2 reviews related work on resource management in satellite communication systems and space information networks, highlighting the gap in existing studies regarding high-priority and time-sensitive services. Section 3 analyzes the key influencing factors of beam-hopping scheduling, including service characteristics, resource constraints, channel conditions, user mobility, and scheduling strategies. Section 4 establishes the system model and formulates the beam-hopping scheduling problem as a system-gain maximization under constraints of fairness, interference, minimum slot allocation, and delay-guarantee for high-priority services. Section 5 details the proposed heuristic beam scheduling algorithm, including the computation of cell weights and costs, the determination of beam-hopping intervals for time-sensitive traffic, and the beam pointing calculation, followed by an illustrative example. The details of the graph reinforcement learning-based beam scheduling approach is proposed in Section 6. Section 7 presents simulation results that evaluate the system gain, user satisfaction, Jain’s fairness index, system overhead and convergence, with a focus on quality-of-service guarantee for high-priority and time-sensitive services. Finally, Section 8 concludes the paper and discusses several directions for future work, such as adaptive beam-hopping pattern design, deep reinforcement learning-based distributed scheduling, and integration with advanced transmission techniques.

## 2. Related Works

In recent years, beam-hopping scheduling for LEO satellites has become a research hotspot in the field of satellite communication. This section reviews the existing literature, summarizes research progress, analyzes key technologies and challenges, and investigates future development directions.

### 2.1. Performance Analysis of Beam Hopping

In [1], the authors analyze uplink performance in beam-hopping satellite systems using discrete-time queuing theory. Two modes (probabilistic and deterministic) are modeled via Markov chains, deriving explicit expressions for throughput, buffer occupancy, packet loss and delay via numerical fixed-point iteration. The key findings of that paper include performance inflection points with beam resource and lower deterministic-mode delay near saturation. However, that study acknowledges several limitations, including the reliance on ideal hopping patterns, the disregard for inter-beam interference, and the assumption that each time slot accommodates at most one packet.

### 2.2. Beam Hopping with Interference Management

Sun et al. [2] proposed the energy-efficient maximal traffic satisfaction beam-hopping (EEMTS-BH) strategy for geosynchronous orbit (GSO) and non-geosynchronous orbit (NGSO) coexistence scenarios, aiming to balance quality of service (QoS) and energy efficiency (EE). They decomposed the joint multi-satellite beam-hopping optimization problem into four sequential sub-problems, including satellite–cell assignment and power allocation. Simulation results showed their strategy outperformed existing ones in traffic satisfaction rate and energy consumption, achieving an optimal trade-off between the two key performance indicators.

The authors in [3] proposes a three-step multi-satellite beam-hopping method for NGSO systems: load balancing via a mixed-integer linear programming (MILP)/greedy approach, single-satellite pattern design using spatially isolated patterns and MILP, and inter-satellite interference avoidance by scheduling patterns with minimum distance penalty. Simulations show a 72.5% load gap reduction and 81.4% satisfaction rate, outperforming seven benchmarks.

The letter [4] proposes a low-complexity multi-satellite beam-hopping algorithm for interference mitigation under tilted beams. It models satellite–cell association (SCA) and BH slot assignment (BHSA) as a dynamic graph coloring problem and solves it via minimum-cost maximum-flow (MCMF) plus tabu search and graph coloring (joint optimization). Simulations on a 10800-satellite constellation show near-optimal performance with far lower complexity than baselines.

The paper [5] proposes throughput-driven beam hopping (TDBH) and satisfaction-rate-driven beam hopping (SDBH) for interference avoidance in integrated satellite–terrestrial networks under given interference patterns. TDBH optimizes throughput with adjustable traffic via relaxation and slot allocation; SDBH maximizes satisfaction rate via genetic algorithm with unserved-time fairness. Simulations show TDBH outperforms random scheduling; SDBH with genetic algorithm (GA) achieves 60.6% average satisfaction, 16.96% higher than genetic algorithm beam hopping (GABH).

Across [3,4,5], common limitations include fixed power allocation, plus algorithm-specific issues: exponential complexity and simplified interference [3], offline pre-solving [4], and pre-known patterns with independent scheduling [5]. Although [2] reports no explicit drawbacks, its sequential decomposition may compromise global optimality. Collectively, these works lack adaptive power control, real-time adaptability, and mutual interference handling, calling for more integrated dynamic frameworks.

### 2.3. Beam Hopping with Precoding

In [6], the authors propose an angle-based multicast user selection, beam selection, and precoding scheme for LEO satellite systems using only elevation and azimuth information, without relying on full channel state information (CSI). A low-complexity greedy algorithm and closed-form precoding are developed. Simulations show performance comparable to CSI-based methods, demonstrating robustness and feasibility for practical beam-hopping satellite communications.

A priority-based Viterbi algorithm (PBVA) is proposed in [7] for beam hopping in LEO satellite systems with hybrid precoding, minimizing transmit power under non-uniform rate demands. Candidate snapshots are pre-filtered using inter-cell distances. Cells are prioritized by demand, and path lengths correspond to Minimum Mean Square Error (MMSE)-based precoding power. A priority-based Viterbi algorithm (PBVA) outperforms the greedy baseline by 2.8 dB and approaches the lower bound within 0.5 dB.

Jointly optimizing linear precoding, beam hopping, and DVB-S2X (Digital Video Broadcasting-Second Generation Extensions) MODCOD (MODulation and CODing) selection for GEO satellites to minimize payload power under user demand constraints is presented in [8]. It relaxes discrete rates and sparsity via fitting and a reweighted l1-norm and proposes a window-based iterative algorithm. Two two-phase methods (heuristic and Deep Neuron Network (DNN)) are also provided. Simulations show power reduction.

In [9], PBVA and Priority-Based Greedy Algorithm (PBGA) for regular and irregular beam-hopping patterns in LEO satellites with hybrid precoding are proposed, minimizing transmit power under rate constraints. Regular patterns use Viterbi on a trellis; irregular patterns use greedy allocation based on demand weights. PBVA outperforms GABH by 2.8 dB, within 0.6 dB of the lower bound. Irregular patterns further improve that by 2 dB.

A dynamic beam illumination framework with selective precoding for GEO beam-hopping systems is proposed in [10], allowing adjacent high-demand beams to be activated and precoded only when needed. Demand constraints are converted to slot counts via expectation maximization (EM)-based estimation. Three binary quadratic programming (BQP) solvers are proposed: Semi-Definition Programming (SDP) relaxation, Multiplier Penalty and Majorization–Minimization (MPMM), and a greedy approach. Simulations show superior demand matching and lower precoding complexity than conventional BH and cluster hopping.

Despite their contributions, the reviewed works share several shortcomings. Most rely on oversimplified channel models (e.g., LOS-only, perfect or even future CSI), which are impractical for dynamic LEO environments. Traffic is typically assumed static, ignoring real-world burstiness. Computational complexity is prohibitive for many methods, particularly those based on window-based iteration or SDP/MPMM, rendering them unsuitable for onboard scheduling. Additionally, the single-user-per-cell/beam abstraction neglects intra-cell interference, and the optimality of heuristic approaches (e.g., PBGA) remains unproven. These gaps highlight the need for a low-complexity, adaptive scheme that accommodates time-varying traffic and imperfect CSI, a need that motivates our proposed framework.

### 2.4. Beam Hopping with Resource Management

In [11], resource allocation in multi-beam LEO satellite integrated sensing and communications (ISAC) systems under full frequency reuse is presented. It proposes a two-stage beam-hopping method based on multi-agent actor–critic reinforcement learning. The first stage optimizes beam patterns to maximize throughput and minimize packet loss while avoiding inter-beam interference. The second stage allocates power to each beam according to traffic demand, improving resource utilization. Simulation results demonstrate effective interference avoidance, increased system throughput, reduced latency, and on-demand power allocation compared to benchmark schemes.

The paper [12] proposes a low-complexity user-density-based beam position design (LCUD-BPD) to minimize beam positions under a radius constraint, and a low-complexity CCI-free beam-hopping design (LCCF-BHD) to minimize time slots under traffic and interference constraints. LCUD-BPD iteratively finds the sparsest user and determines its best beam position (BP) via a user selection and smallest radius algorithm. Duplicated user removal further reduces BP radius. LCCF-BHD prioritizes large-radius or high-demand BPs. Simulations show near-optimal performance with 99.9% complexity reduction.

The article [13] addresses dynamic beam hopping and resource allocation for NGSO satellite systems with non-uniform traffic. Using Lyapunov optimization, it minimizes a per-slot cost that includes the capacity–demand gap and energy/handover penalty. The online Long-Term Joint Optimization for Beam Hopping, Bandwidth Allocation and Power Control (LO-BBP) algorithm combines matching theory (for beam hopping and bandwidth) with convex approximation (for power control). A theoretical analysis shows an [O(1/V),O(V)] trade-off between cost and queue backlog. Simulations demonstrate reduced backlog and energy versus benchmarks.

The paper [14] maximizes energy efficiency (EE) in LEO beam-hopping downlink under minimum offered-capacity-to-demand ratio (OCDR) constraints. It transforms the multi-slot problem into per-slot subproblems: power allocation (iterative Karush–Kuhn–Tucker (KKT)-based) and user selection (priority-based for EE maximization). Simulations show the proposed Energy-Efficient User Selection–Energy-Efficient Power Allocation (EEUS-EEPA) outperforms baselines in EE and OCDR.

In [15], the authors jointly optimize beam scheduling and power allocation in LEO beam-hopping systems to minimize offer–demand mismatch. Beam scheduling is modeled as an exact potential game with Nash equilibrium reached via best response, while power is optimized via a penalty interior-point method. Multiple sink nodes per beam position are considered. Simulations show joint beam scheduling and power optimization beam hopping (JBSPO-BH) outperforms greedy, Round-Robin, Max-SINR (Signal-to-Interference-plus-Noise Ratio), and GA-based schemes in throughput and fairness with lower complexity.

In [16], a mixed-integer linear programming (MILP) framework is proposed to jointly optimize illumination, bandwidth, and power for LEO beam-hopping systems, minimizing unserved capacity (UC), extra-served capacity (EC), and time-to-serve (TTS). A time-split variant reduces complexity by solving sequential sub-windows. Simulations show time-split MILP outperforms demand-based and genetic algorithms: UC improves by 22% (reduced) and 6.5% (enlarged), TTS halves.

The authors in [17] propose multi-satellite dynamic beam coverage–beam hopping (MSDBC-BH) for dual-layer LEO constellations, achieving two-dimensional load balancing via dynamic beam coverage (cell splitting based on traffic density) and game-matching-based cell-satellite assignment. Simulations show beam load gap reduced from 0.0862 to 0.0129, satellite load gap reduced by 93.85%, and throughput improved by 28.90% over random BH (R-BH).

In [18], the authors propose an non-orthogonal multiple access (NOMA)-based collaborative beam hopping scheme for LEO satellites. It designs hopping patterns with interference threshold distance, pairs cell-edge user (CEU) with cell-center user (CCU) into NOMA clusters using SCA-based power allocation, and applies Q-Learning for carrier selection. Simulations show NOMA outperforms time-division multiple access (TDMA) in rate and outage probability.

In [19], the authors propose a cognitive LEO beam-hopping satellite–terrestrial network with underlay spectrum sharing. It introduces power allocation competition (PAC) for satellite pattern/power optimization, dynamic-ratio threshold (DRT) for reliable SST detection, and adaptive resource adjustment (ARA) for terrestrial CCI suppression. PAC complexity is far lower than Particle Swarm Optimization (PSO)/GA. Simulations validate QoS improvement and CCI control.

In [20], LDCB-MSHBRA, a time-slicing-based dynamic resource allocation method for multi-LEO multi-beam beam hopping is proposed. It jointly optimizes time, frequency, and power while balancing load and adapting to coverage changes. Lagrangian duality and one-way search are used. Simulations show 100% QoS satisfaction, 28.15% and 19.56% lower average packet delay than two baselines, and improved throughput. Limitations include small-scale validation (three satellites), fixed beam positions, and no inter-satellite links.

The paper [21] proposes a tabu search-based spatio-temporal beam-hopping resource allocation strategy for LEO satellite communications. It maximizes user-demand satisfaction under interference constraints using adaptive tabu tenure, greedy initialization, and simulated annealing acceptance. Simulations show 17.2% throughput and 11.7% satisfaction improvement over greedy schemes. Limitations include single-satellite scope, simplified traffic/channel models, and stationary user assumption.

Despite these advances, several common limitations persist across the reviewed resource management schemes, including oversimplified channel models (LOS-only, static, or perfect/future CSI) and time-invariant traffic assumptions, which ignore real-world dynamics. Computational complexity is prohibitive for MILP, KKT, and game-theoretic approaches, especially in large-scale constellations. Many adopt narrow abstractions (e.g., single user per cell, fixed power, single satellite), neglecting interference and coordination. Heuristics and Q-learning lack scalability and optimality guarantees; reliance on commercial solvers, perfect CSI, or long-term statistics further hinders deployability. These gaps motivate our low-complexity, adaptive framework.

### 2.5. Beam Hopping with Machine Learning

A multi-agent deep reinforcement learning (DRL) framework for joint beam pattern and bandwidth allocation in GEO beam-hopping systems is presented in [22]. Each beam is split into two agents (illumination and bandwidth) to reduce action space. Agents share global state and reward, trained via independent DDQN. Simulations show throughput gains of 10∼32% over baselines and improved delay fairness. Generalization beyond training traffic range is demonstrated.

The paper [23] addresses joint power and beam scheduling in LEO satellite Internet of Things (IoT) with beam hopping. It decomposes the multi-objective problem into convex power optimization and DRL-based scheduling. The proposed action-masking multiobjective double deep Q-network (AMM-DDQN) uses Chebyshev scaling and action masking to handle multiple objectives and large action spaces. Simulations show real time delay reduced by 4.42∼22.51% over baselines, with improved throughput and fairness.

The authors in [24] propose a cooperative multi-agent Value-Decomposition Networks with Dueling Double Deep Q-Learning Network (VDN-D3QN) framework for dynamic beam hopping in LEO satellite systems with non-uniform traffic. Centralized training and distributed execution avoid inter-satellite information exchange. Each agent controls one satellite, and VDN decomposes the joint Q-value. The multi-objective reward balances throughput and delay fairness. Simulations show improved performance over baselines.

In [25], the letter proposes DeepBeam, a DRL-based joint optimization of beam-hopping scheduling and coverage control for multibeam GEO satellites. It dynamically selects each beam’s center cell and coverage radius to maximize throughput. To reduce action space, it trains a single agent and applies it to all beams with pseudo-state updates. Simulations show 20∼34% throughput gain and 10∼30% lower packet loss versus heuristics.

A two-stage satellite–terrestrial coordinated multi-satellite beam hopping framework is proposed in [26]. Network operation control center (NOCC) solves long-term cell-satellite association via a greedy + iteration approach to balance load; each satellite uses offline-trained QMIX (centralized training, distributed execution) for real-time traffic-driven scheduling with spatial isolation and parameter sharing. Simulations show a 70% load-gap reduction, 50% delay reduction, <4 ms average delay, and <1 ms inference time.

In [27], the authors propose DeepMBS, a parameterized DRL framework for joint beam hopping (discrete) and power allocation (continuous) in multi-beam satellite systems. It extends a deep Q-network (DQN) with a parameterized action space, where a policy network outputs continuous power for each discrete cell choice. An experience filtering mechanism accelerates convergence. Simulations show 14.3∼57.6% throughput gain and 6.9∼38.9% energy efficiency improvement over baselines.

A user-level dynamic beam hopping scheme for LEO satellite networks in [28], jointly optimizing beam pattern and access control based on real-time per-user traffic. A user-oriented Markov Decision Process (MDP) converts the long-term problem to short-term Q-value maximization. A deep reinforcement learning-assisted enhanced genetic (DRL-EG) algorithm estimates Q-values via Sarsa and solves scheduling via an enhanced genetic algorithm. Simulations show throughput and capacity gains over cell-level baselines.

The authors in [29] propose multiagent proximal policy optimization–beam pattern and resource allocation (MAPPO-BPRA) for multi-LEO satellite systems, jointly optimizing beam pattern, bandwidth, power, and precoding to maximize minimum traffic satisfaction. Each satellite is an agent with hybrid actions (discrete beam/bandwidth, continuous power/precoding), trained via centralized critic with decentralized execution. Simulations show superior satisfaction and throughput over other approaches.

An attention-based multi-agent communication framework for NGSO multi-satellite beam hopping is studied in [30]. It decouples the problem into user-level load balancing and real-time BH scheduling via MAPPO with self-attention and periodic inter-satellite communication. Self-attention handles dynamically visible cells; communication via learned attention weights enables topology-adaptive coordination. Simulations show 17% throughput gain over MLP-based MADRL with <0.01% ISL overhead.

Despite the promise of DRL-based approaches, common limitations persist across these works. Most rely on small-scale simulations (e.g., 2–3 satellites, 35 beams), fixed power allocation, and oversimplified channel models (e.g., LOS-only or perfect CSI), which fail to reflect real-world LEO dynamics. Many schemes adopt single-satellite scenarios, neglecting inter-satellite coordination, or suffer from limited generalization beyond training traffic ranges. Action space simplifications (e.g., discrete radii, independent agents without explicit coordination) and two-stage suboptimality further constrain performance. Online DRL training overhead and reliance on CVX-based optimization onboard remain impractical. Collectively, these gaps highlight the need for scalable, generalizable, and coordination-aware scheduling frameworks.

### 2.6. Beam Hopping with Beamforming

The paper [31] proposes a hybrid beamforming (HBF) design for beam-hopping LEO SatComs to maximize sum-rate under illumination and rate constraints. It first designs illumination patterns via random search and fractional programming (FP) under fully digital beamforming (FDBF), then optimizes HBF by generating candidate analog beamformers and solving digital ones via FP. Simulations show HBF approaches FDBF performance. Limitations include random analog beamformers, single illumination per beam position, and time-invariant channel assumption.

In [32], hybrid beamforming for beam-hopping LEO SatComs to maximize sum-rate under HBF and rate constraints is investigated. It proposes FDBF-IPRS (FDBF and illumination pattern random search) and FDBF-IPAO (FDBF and illumination pattern alternating optimization) to design illumination patterns and fully digital beamformers, then HBF-AM (HBF alternating minimization) to design hybrid beamformers. IPAO significantly reduces complexity versus IPRS. Simulations show HBF approaches FDBF performance. Limitations include single-illumination per beam position and time-invariant channels.

The hybrid beamforming schemes rely on random analog beamformers, single illumination per beam position, and time-invariant channel assumptions, which fail to capture the dynamic nature of LEO satellite environments. These simplifications limit practical applicability and motivate the need for more adaptive and robust beamforming designs.

### 2.7. Beam Hopping with Multiple Access

The paper [33] jointly designs beam hopping and multiple access for integrated satellite–terrestrial communication network (ISTCN). It proposes cognitive radio-beam hopping (CR-BH), which schedules beams based on both traffic demand and available spectrum, and adaptive dynamic multiple access (ADMA), which adaptively adjusts the overload factor. Multi-satellite joint service handles beam-edge interference. Simulations show CR-BH + ADMA outperforms conventional BH and fixed multiple access. However, the proposed method relies on a simplified priority model, idealistic spectrum sensing, and an underdeveloped optimization strategy, which collectively limit its practical applicability.

In [34], the authors propose BH-GFRA, an intelligent beam-hopping grant-free random-access architecture for LEO IoT. It integrates random, group and orthogonal resource hopping with spatio-temporal activation optimization, and derives analytical models for collision, delay, success rate and BLER. Simulations show lower delay and better BLER than two-step RA under high activity. Limitations include idealized assumptions, limited security analysis, and small-scale validation.

Despite significant contributions across various domains—including performance analysis, interference management, precoding, resource allocation, machine learning, beamforming, and multiple access—the reviewed works share several common limitations. Most rely on oversimplified channel models (e.g., LOS-only, perfect or future CSI) and static traffic assumptions, failing to capture real-world LEO dynamics. Computational complexity remains prohibitive for many optimization-based schemes, while DRL approaches suffer from small-scale simulations, limited generalization, and fixed power allocation. Many adopt narrow abstractions such as single-user-per-cell, single-satellite scenarios, or independent optimization of individual resources, neglecting holistic network-wide coordination. These gaps collectively motivate the need for a unified, low-complexity, and adaptive resource management framework for multi-layer satellite communication systems.

## 3. Influencing Factors of Beam-Hopping Scheduling

The beam-hopping scheduling of satellite communication systems is affected by multiple factors, which need to comprehensively consider physical environment, business requirements, system resources, scheduling strategies and other aspects.

### 3.1. Business Requirement Characteristics

Spatio-temporal distribution heterogeneity. Traffic demand varies significantly across time and geography, requiring real-time awareness of demand hotspots.

Service type and QoS requirements. Voice, video, IoT and broadband services have different latency, jitter, loss, bandwidth and reliability requirements. High-QoS services must be prioritized.

Traffic burstiness. Temporary events can cause sudden demand surges, requiring rapid beam reallocation.

### 3.2. System Resource Constraints

Total power constraint. Satellite power is limited so that scheduling must balance beam activation and power allocation.

Beamforming capability. Phased-array or multi-beam antennas can form only a limited number of simultaneous beams.

Spectrum resources. Scarce spectrum requires tight reuse and effective co-channel/adjacent-channel interference control.

On-board processing and switching capability. Switching matrix capacity and baseband processing power limit forwarding and scheduling complexity.

Beam switching time. Pointing, frequency and timing switching take milliseconds, and frequent switching reduces effective communication time.

### 3.3. Channel Conditions and Propagation Environment

Path loss and atmospheric attenuation. Free-space loss, rain, clouds and atmospheric absorption degrade link quality. Users with poor channels may need more resources.

Multipath fading and shadowing effect. Mobile and urban scenarios cause fast or slow fading, and scheduling should be robust or use channel state information.

Doppler shift. High-speed satellites and terminals cause frequency offset, which requires compensation.

### 3.4. User Characteristics and Mobility

User distribution density. Dense areas require smaller beams or more frequent service, affecting beam size and dwell time.

User mobility. Moving terminals and LEO/MEO satellites cause rapid relative position changes. Beam scheduling must predict user positions and manage beam handover.

Terminal capabilities. Transmit power, antenna gain and modulation/coding affect uplink quality and achievable rate.

### 3.5. Scheduling Strategies and Algorithms

Optimization objectives. Throughput, fairness, QoS, latency and power consumption may lead to different scheduling strategies.

Scheduling cycle and time slot structure. Update frequency and fixed/variable slot duration affect response speed and resource flexibility.

Information acquisition and prediction. Queue status, channel state, user position and demand prediction accuracy directly affect scheduling performance.

Algorithm complexity. Optimal scheduling is NP-hard. Hence, practical systems must balance performance and real-time complexity using heuristics or learning-based approaches.

Interference management mechanism. Coordination with power control, beamforming nulling and interference coordination is key to system capacity.

### 3.6. Space-Ground Collaboration and Network Architecture

Feeder link resources. Feeder bandwidth is a bottleneck, and BH downlink scheduling must coordinate with feeder-link allocation.

Inter-satellite links. ISLs enable data relaying, and affect routing and downlink beam scheduling [35].

Centralized vs. distributed scheduling. Ground-centralized decisions have long control latency, while on-board distributed decisions face limited computing power.

In summary, efficient BH scheduling must jointly consider and balance dynamic business demands, strict on-board resource constraints, time-varying channels, user mobility and scheduling algorithms.

## 4. System Model

Figure 1 gives the LEO beam-hopping satellite communication system. The satellite illuminates *K* hexagonal cells on the ground via *J* reconfigurable beams, where different colors represent different beams, enabling flexible resource allocation and coverage.

Considering the above influencing factors, this paper formulates the beam scheduling problem of beam-hopping satellite communication systems for high-priority and delay-sensitive services as a system-gain maximization optimization problem under the aforementioned constraints.

**Definition** **1.** *From the perspective of revenue and cost, the system gain of cell k is defined as the difference between the revenue obtained from serving users and the cost incurred within one beam-hopping cycle in a beam-hopping satellite communication system, which can be expressed as:*

(1) $$g_{k}=r_{k}-c_{k}$$

*where $r_{k}$ is the revenue of cell k, which is proportional to its service weight $w_{k}$, i.e., $r_{k}=\eta w_{k}$, where η>0 is a unit revenue coefficient. For simplicity, η=1 is adopted in the simulations, and $c_{k}\geq 0$ is the cost of serving cell k, including power consumption, bandwidth occupation, beam switching and signaling overhead.*

The degree of user-demand satisfaction is mainly reflected by the traffic volume generated by serving users and the level of meeting users’ QoS requirements. The resource usage cost mainly refers to the estimated cost of satellite resource consumption, including resource overhead such as power and channel resources to meet service demands, as well as signaling overhead caused by satellite–ground interaction, beam switching and satellite handover. Based on this definition, a beam scheduling scheme achieves desirable performance if it enables the system to serve more users with better quality and achieve higher traffic volume through better channel conditions and lower system overhead within a beam-hopping period. Therefore, under the constraints of inter-beam interference mitigation, user fairness and minimum time slot allocation, this paper maximizes the system gain on the premise of guaranteeing high-priority and delay-sensitive services.

For beam-hopping scheduling, the final form of the scheduling result is a set of triples {t,j,k}, indicating that beam *j* illuminates spot *k* in time slot *t*. Accordingly, this paper formulates the beam-hopping scheduling problem for LEO satellite networks serving high-priority and delay-sensitive services as the following optimization problem,

(2) $$max\sum_{t}\sum_{j}\sum_{k}x_{jk}\left(t\right)\cdot g_{k}$$

s.t.

(3) $$\sum_{t}\sum_{j}x_{jk}\left(t\right)\leq P_{k}T,\forall t\in T,\forall j\in J$$

(4) $$\sum_{t}\sum_{k}x_{jk}\left(t\right)\leq J$$

(5) $$\theta_{mn}\left(t\right)\geq \theta_{c},\forall t\in T,\forall m,n\in K$$

(6) $$\sum_{t}\sum_{j}x_{jk}\left(t\right)\geq \tau^{\prime }$$

(7) $$S_{k}\left\{t\right\}-S_{k}\left\{t-1\right\}\leq \Delta t_{k},1\leq t\leq \left|S_{k}\right|$$

(8) $$x_{jk}\left(t\right)=\left\{0,1\right\}$$

(9) $$c_{k}\geq 0$$

(10) $$r_{k}\geq c_{k}$$

In the above formulation, Constraint (3) is the fairness constraint, which ensures that cells with larger traffic volume and higher gain can be allocated more time slots. Without this constraint, a greedy scheduler would continuously serve the cells with the highest weights, leading to severe unfairness. Equation (4) maintains the practically available satellite beams and ensures that any cell is illuminated by at most one beam in a given time slot. Constraint (5) indicates that the angle between any two beams is greater than the co-channel interference angle threshold between beams. Constraint (6) is the minimum time slot constraint to guarantee the minimum number of access time slots for a cell to the satellite within one beam-hopping period, where $\tau^{\prime }$ is the minimum number of time slots allocated to each cell in a beam hopping cycle. Together with constraint (3), Constraint (6) provides both an upper and a lower bound on the resource allocated to each cell, thereby balancing system gain and fairness. Constraint (7) ensures the priority beam coverage and scheduling for high-priority and delay-sensitive services, so that they can be completed within their QoS time requirements, where $S_{k}\left\{t\right\}$ is the set of time slots for lighting cell *k* in a beam-hopping cycle before time slot *t*. Constraint (8) specifies that the parameter is a binary variable (0 or 1). Constraint (9) stipulates that the communication cost and overhead are non-negative. Constraint (10) requires that the system gain of scheduling a beam to illuminate cell *k* is not less than its cost or expense; otherwise, it is regarded as an invalid scheduling.

## 5. Heuristic Beam Scheduling

When calculating the weight of each cell, the importance, delay requirements and channel conditions of non-high-priority and non-delay-sensitive services within the cell are mainly considered, where the channel conditions include link loss and multipath effects. When accounting for the communication cost of each cell, the energy consumption and signaling overhead during service communication are mainly taken into account.

In this paper, the weighting method for cells is given by the following equation,

(11) $$w_{k}=\alpha_{k}^{1}\cdot min\left\{v_{k},C\right\}+\frac{\alpha_{k}^{2}}{\tau_{k}}+\alpha_{k}^{3}\cdot h_{k}$$

where $v_{k}$ denotes the traffic volume to be served in cell *k*, and $\alpha_{k}^{1}$ is the traffic weight determined by the traffic importance. $\tau_{k}$ represents the average delay requirement of the traffic to be served in cell *k*, and $\alpha_{k}^{2}$ is the delay weight determined by the delay significance. $h_{k}$ stands for the average channel response gain from the satellite to cell *k*, and $\alpha_{k}^{3}$ is the channel weight determined by the significance of transmission efficiency brought by channel gain. The channel response gain mainly takes into account path loss, multipath fading, rain attenuation and other factors.

The cost calculation mainly takes into account the service bandwidth, energy consumption and signaling overhead, and its calculation method is given by the following formula,

(12) $$c_{k}=\beta_{k}^{1}\cdot b_{k}+\beta_{k}^{2}\cdot e_{k}+\beta_{k}^{3}\cdot o_{k}$$

where $b_{k}$ denotes the bandwidth required by the satellite to provide services for cell *k*, and $\beta_{k}^{1}$ is the bandwidth weight determined by bandwidth scarcity or spectrum pricing, i.e., the spectrum cost. $e_{k}$ represents the energy consumed by the satellite to serve cell *k*, and $\beta_{k}^{2}$ is the energy weight determined according to the remaining energy of the satellite. $o_{k}$ stands for the signaling overhead generated by scheduling beams to serve cell *k*, and $\beta_{k}^{3}$ is the overhead weight determined by the magnitude of signaling overhead. The signaling overhead mainly includes beam-hopping pattern distribution, beam switching and satellite switching overhead.

### 5.1. Beam Interval Calculation for High-Priority and Delay-Sensitive Services

To meet the requirements of high-priority and low-latency services, the minimum illumination interval $\Delta t_{k}$ for cell *k* is calculated as follows,

(13) $$\Delta t_{k}=\frac{R_{k}^{r}}{R_{j}^{\prime }}$$

where $R_{k}^{r}$ denotes the generation rate of high-priority and time-sensitive data within cell *k*, and $R_{j}^{\prime }$ represents the maximum transmission rate that can be provided by beam *j*.

Therefore, for high-priority and low-latency service requirements, the maximum number of beam-hopping intervals for illuminating cell *k* is given by,

(14) $$n_{k}^{r}=\left\{\begin{array}{cc} \lfloor \frac{R_{k}^{r}/R_{j}^{\prime }}{T_{h}}\rfloor , & \frac{R_{k}^{r}/R_{j}^{\prime }}{T_{h}}\geq 1\\ 0, & 0<\frac{R_{k}^{r}/R_{j}^{\prime }}{T_{h}}<1 \end{array}\right.$$

where $T_{h}$ denotes the beam-hopping time interval.

### 5.2. Calculation of Beam Transmit Azimuth and Elevation Angles

The algorithm and procedure for calculating the transmit azimuth and elevation angles of the beam are as follows:

1. Convert the longitude, latitude and altitude of the satellite and the center of the cell to Cartesian coordinates in the Earth-centered Earth-fixed (ECEF) frame, where the satellite coordinate vector is denoted as $r_{eS}^{e}$ and the beam center coordinate vector as $r_{en}^{e}$.
2. The vector pointing from the cell center to the satellite in the Earth-fixed frame (e-frame) is denoted as $r_{nS}^{e}=r_{eS}^{e}-r_{en}^{e}$.
3. Transform this vector into the local north–east–up (n-frame) with the cell center as the origin, and the corresponding coordinate is denoted as $r_{nS}^{n}=C_{e}^{n}r_{nS}^{e}$, where $L_{b}$, $\lambda_{b}$ and $h_{b}$ are the latitude, longitude and altitude of the cell center, respectively. The n-frame is defined with its origin at the beam center, the x-axis pointing to true north, the y-axis pointing east, and the z-axis along the normal of the local Earth ellipsoid, where
  (15) $$C_{e}^{n}=\left[\begin{array}{ccc} -\sin L_{b}\cos\lambda_{b} & -\sin L_{b}\sin\lambda_{b} & \cos L_{b}\\ -\sin\lambda_{b} & \cos\lambda_{b} & 0\\ -\cos L_{b}-\cos\lambda_{b} & -\cos L_{b}\sin\lambda_{b} & -\sin L_{b} \end{array}\right]$$
4. The vector obtained above is denoted as $r_{nS}^{n}={\left[x_{nS}^{n},y_{nS}^{n},z_{nS}^{n}\right]}^{T}$.
5. The corresponding elevation angle and azimuth angle can be obtained as:
  (16) $$\left\{\begin{array}{c} El_{bs}=\arctan\frac{-z_{nS}^{n}}{\sqrt{{\left(x_{nS}^{n}\right)}^{2}+{\left(y_{nS}^{n}\right)}^{2}}}\\ Az_{bs}=\arctan\left(y_{nS}^{n},x_{nS}^{n}\right) \end{array}\right.$$
  where $El_{bs}$ is the elevation angle from the beam center to the satellite, and $Az_{bs}$ is the azimuth angle from the beam center to the satellite. The azimuth angle is defined to be positive when rotating eastward from true north and negative when rotating westward.
6. The beam pointing azimuth from the satellite to the center of the cell is obtained via angle transformation,
  (17) $$Az_{sb}=\left\{\begin{array}{cc} Az_{bs}, & Az_{bs}\geq 0\\ 360-Az_{bs}, & others \end{array}\right.$$
7. The geocentric angle between the beam center and the satellite is calculated using their ECEF coordinates,
  (18) $$\xi =\arctan\left(\frac{\left|r_{eS}^{e}\times r_{en}^{e}\right|}{r_{eS}^{e}\cdot r_{en}^{e}}\right)$$
8. The elevation angle of the satellite with respect to the beam center is derived from the geocentric angle,
  (19) $$El_{sb}=90-\xi$$
9. Given the cell *m* and *n*, the outgoing unit vectors ${\vec{e}}_{sb}^{m}$ and ${\vec{e}}_{sb}^{n}$ from the satellite to each beam center are obtained according to the calculated beam transmit elevation angles and azimuth angles from the satellite to the beam centers, respectively, as described above.
10. The angle between the two beams is finally derived as
  (20) $$\theta_{k_{m}k_{n}}=\arctan\left(\frac{\left|{\vec{e}}_{sb}^{m}\times {\vec{e}}_{sb}^{n}\right|}{{\vec{e}}_{sb}^{m}\cdot {\vec{e}}_{sb}^{n}}\right)$$

### 5.3. Heuristic Algorithm for Beam Scheduling

For beam-hopping scheduling targeting high-priority and time-sensitive services, the satellite must first meet the requirements of such traffic. By calculating the required beam-hopping time interval, the slots for cells containing these services can be pre-scheduled or prioritized. Subsequently, the beam-hopping patterns for other services within these cells can be optimally scheduled. Here, we assume that the required number of slots with high-priority/time-sensitive service does not exceed the total number of available ones.

In summary, the heuristic computation of the maximum-gain beam-hopping pattern for high-priority and time-sensitive services is presented in Algorithm 1. The algorithm first schedules high-priority and time-sensitive services to prioritize the service demands of cells carrying such traffic; heuristic approaches are then adopted to schedule the remaining services. To achieve a more balanced workload distribution across beams, the beam indices can be adjusted either periodically or aperiodically.

**Algorithm 1** Heuristic beam scheduling algorithm (HBSA)
**Require:** Longitude, latitude, and altitude of the satellite; the longitude, latitude, and altitude of the center of each cell within the service area; the traffic distribution of services; the bandwidth and frequency band utilized by the satellite; and the maximum number of available beams, and T, J, K, R, P, $K_{d}$, $T_{d}$.**Ensure:** The lighted cells and corresponding assigned beams in each time slot constitute the beam-hopping scheduling results H.
  1:The cells carrying high-priority and low-latency services are determined based on parameter $K_{d}$.  2:Based on the service characteristics of the cells indicated by $K_{d}$, the number of beam-hopping intervals $T_{d}$ for each cell is calculated.  3:Calculate the proportion of service volume in each cell to the total service volume in the satellite service area.  4:**for** t∈T **do**  5:    **for** j∈J **do**  6:      **for** $k\in K_{d}$ **do**  7:         Obtain the maximum value $t_{max}^{k}$ of time slots allocated to cell *k* from the set H.  8:         **if** $\left(t-t_{max}^{k}\right)\geq T_{d}\left(k^{\prime }\right)$ and $K_{d}\left(k^{\prime }\right)==k$ and beam *j* is idle at slot *t* **then**  9:            H←H∪{t,j,k}.10:            Update $K_{d}$, $T_{d}$ and R.11:         **else**12:            Pass.13:         **end if**14:      **end for**15:    **end for**16:
**end for**
17:**for** t∈T **do**18:    $R_{temp}=R$.19:    **for** j∈J **do**20:      **while** $R_{temp}\neq \emptyset$ **do**21:         Select the cell $k_{max}$ with the heavest traffic volume.22:         **if** the slots assigned to cell $k_{max}$ are not larger than $P(k_{max})T$ and $R(k_{max})>0$         **then**23:            **if** the CCI between $k_{max}$ and the other cells that have been assigned beams            in slot *t* is less than interference threshold $\theta_{c}$ and $\left\{t,j,k_{max}\notin H\right\}$ or the time            elapsed since the last illumination of cell *k* is greater than $▵t_{k}$ **then**24:            Assign slot *t* and beam *j* to cell $k_{max}$.25:            Set the traffic volume corresponding to cell $k_{max}$ in $R_{temp}$ to zero.26:            Update the remaining traffic volume of cell $k_{max}$ in R.27:            Break.28:            **else**29:            Set the traffic volume corresponding to cell $k_{max}$ in $R_{temp}$ to zero.30:            **end if**31:         **else**32:            Set the traffic volume corresponding to cell $k_{max}$ in $R_{temp}$ to zero.33:         **end if**34:      **end while**35:    **end for**36:
**end for**



Constraint handling in HBSA: Algorithm 1 follows a feasibility-preserving greedy construction. Lines 4–16 first pre-schedule cells carrying high-priority and time-sensitive services, thereby enforcing Constraint (7) and guaranteeing the minimum service for these cells. Lines 17–36 then allocate the remaining time slots to other cells. At each step, the candidate cell $k_{max}$ is accepted only if: (i) its allocated slots do not exceed $P(k_{max})T$ (Constraint (3)); (ii) its remaining traffic $R(k_{max})>0$ and its minimum reservation is considered (Constraint (6)); (iii) the CCI with already scheduled cells is below $\theta_{c}$ (Constraint (5)); (iv) beam *j* is idle at slot *t* (Constraint (4)); and (v) its gain is not less than its cost (Constraint (10)). If a candidate violates any of these conditions, it is removed from $R_{temp}$, and the algorithm proceeds to the next candidate. If no feasible cell remains, the beam is left idle. The binary Constraint (8) and the non-negative cost Constraint (9) are satisfied by definition. Therefore, every scheduling result in H is feasible.

The heuristic principle of HBSA is a two-stage, feasibility-preserving greedy construction: (i) priority-driven pre-scheduling for high-priority and time-sensitive cells, which enforces their maximum tolerable service interval; and (ii) greedy maximum-weight-first allocation for the remaining resources, where each cell is selected only if it satisfies the fairness, beam, interference, minimum-slot and profitability constraints. This principle directly corresponds to lines 4–16 and 17–36 of Algorithm 1, respectively.

### 5.4. Performance Analysis

Let $R_{k}^{r}$ denote the generation rate of high-priority data in cell *k*, and let $R_{j}^{r}$ be the maximum service rate that a single beam can provide. To ensure system stability (i.e., to prevent the queue from growing without bound), the necessary and sufficient condition is

(21) $$\rho_{k}\leq 1$$

where $\rho_{k}=\frac{R_{k}^{r}}{R_{j}^{r}}$.

Derivation: From Equations (13) and (14), the maximum tolerable beam-hopping interval is obtained as $▵t_{k}$, and the normalized demand is given by

(22) $$n_{k}^{r}=\left\lfloor \frac{▵t_{k}}{T_{h}}\right\rfloor$$

Performance upper bound (delay bound): In the proposed algorithm, high-priority cell *r* is served at least once every $n_{k}^{r}+1$ time slots (enforced by line 8 of Algorithm 1). Assuming a first-in-first-out (FIFO) queuing discipline, the maximum queuing delay $D_{max}$ of a data packet satisfies:

(23) $$D_{max}\leq \left(n_{k}^{r}+1\right)\cdot T_{h}+T_{prop}$$

where $T_{prop}$ is the one-way propagation delay of the LEO satellite link (typically 5–15 ms). Substituting the expression for $n_{k}^{r}=\left\lfloor \left(R_{k}^{r}/R_{j}^{r}\right)/T_{h}\right\rfloor$ into the inequality for $D_{max}$ yields

(24) $$D_{max}\leq T_{h}\cdot \left\lfloor R_{k}^{r}/\left(R_{j}^{r}\cdot T_{h}\right)\right\rfloor +T_{h}+T_{prop}$$

which, in turn, can be bounded further given the definition of $n_{k}^{r}$. This demonstrates that, as long as the algorithm forcibly inserts service for high-priority cells, the worst-case delay is strictly constrained to the millisecond level, thereby providing a deterministic guarantee for time-sensitive services.

Owing to the co-channel interference constraint (Constraint (5)), the cells can be mapped to a conflict graph, where the vertices represent the cells, and edges connect pairs of cells whose angular separation is less than $\theta_{c}$. The scheduling problem is then equivalent to finding a maximum weighted independent set (MWIS) on this conflict graph in each time slot.

The MWIS problem is NP-hard in general graphs. In the proposed algorithm, a greedy maximum-weight-first strategy is adopted in lines 21∼24.

Proof of the performance bound: Let OPT denote the total system gain of an optimal solution in a given time slot, and let ALG denote the total gain obtained by the greedy solution. Let the first vertex chosen by the greedy algorithm be $v_{1}$, with weight $w_{v_{1}}$. Since the greedy choice is the one with the globally maximum weight, it at least eliminates all conflicting neighbours of $v_{1}$ that are present in the optimal solution. Although the classical greedy algorithm for MWIS has a worst-case bound of 1/▵ (where ▵ is the maximum degree), the geometric constraint expressed in Equation (5), combined with the fact that the 3 dB beamwidth of the satellite antenna is finite, implies that the maximum number of conflicting neighbours ▵ for any cell is bounded by the ratio of the satellite coverage area to the minimum beam area (which is a constant). Consequently, the per-slot system gain of the proposed algorithm is bounded below as follows:

(25) $$ALG_{i}\leq \frac{1}{▵+1}\cdot OPT_{i}$$

Since ▵ is determined by the physical antenna parameters (e.g., beamwidth), it remains unchanged when the number of beams *J* increases while the coverage area is fixed. This indicates that the proposed algorithm maintains a favourable approximation performance even in large-scale constellations, while its complexity is far lower than that of the brute-force MWC algorithm.

Our proposed algorithm (HBSA) consists primarily of three nested loops, corresponding to the number of time slots *T*, the number of beams *J*, and the number of cells *K* (in the worst case, the while loop in line 20 iterates over all cells).

The complexity of HBSA is derived step by step based on the actual loops and operations in Algorithm 1.

Stage 1: Priority-driven pre-scheduling (lines 4–16).

The outer loop over time slots runs *T* times, the middle loop over beams runs *J* times, and the inner loop over high-priority cells runs $|K_{d}|$ times. For each iteration, the algorithm obtains the last service slot $t_{\max}^{k}$ and checks the deadline condition. If $t_{\max}^{k}$ is maintained in a simple array, each check takes O(1). Therefore, the complexity of Stage 1 is

(26) $$O(T\times J\times |K_{d}|)\leq O\left(T\times J\times K\right)$$

Stage 2: Greedy allocation for remaining services (lines 17–36). For each time slot t∈T, the algorithm initializes a temporary set $R_{\mathrm{temp}}=R$, which takes O(K). Then, for each beam j∈J, it repeatedly selects the cell $k_{\max}$ with the heaviest traffic volume from $R_{\mathrm{temp}}$. A key observation is that $R_{\mathrm{temp}}$ is shared across all beams within the same time slot. Once a cell is either scheduled or removed from $R_{\mathrm{temp}}$, it is never considered again in that slot. Therefore, for a fixed *t*, the total number of while-loop iterations over all beams is at most *K*.

The cost of selecting $k_{\max}$ depends on the data structure used. If a linear scan over $R_{\mathrm{temp}}$ is used, each selection costs $O(|R_{\mathrm{temp}}|)$, and the total cost per time slot is $O(K^{2})$. If the cells are pre-sorted by weight or maintained in a priority queue, each selection costs O(logK) and each removal costs O(logK). Then, the total cost per time slot is O(KlogK+JlogK).

In our implementation, we adopt the priority-queue approach. Therefore, for each time slot, the complexity of Stage 2 is

(27) O(KlogK+JlogK)

Since the outer loop over time slots runs *T* times, the total complexity of Stage 2 is

(28) O(T×(KlogK+JlogK))

Combining Stage 1 and Stage 2, and noting that J≤K in typical LEO beam-hopping systems, the overall complexity of HBSA is

(29) O(T×J×K)+O(T×(KlogK+JlogK))

If the simpler linear-scan implementation is used, the worst-case complexity becomes $O(T\times J\times K+T\times K^{2})$. Since *J* and *K* are of the same order in practice, this is still polynomial and far lower than the exponential complexity $O(2^{K})$ of the MWC algorithm.

For comparison, the Round-Robin scheme has a complexity of O(T×J) (as it does not involve weight computation), while the MWC algorithm, which is based on clique search, incurs a worst-case complexity of $O(2^{K})$, i.e., exponential in the number of cells.

The high mobility of LEO satellites renders frequent signaling interactions a performance bottleneck. The proposed algorithm adopts a centralized pre-computation approach, in which the hopping pattern is calculated at the ground station; control signaling of only $T\times J\times log_{2}K$ bits is transmitted once at the beginning of each scheduling cycle, as only the triplets {t,j,k} need to be broadcast. In contrast to the online deep reinforcement learning scheme in [24], which requires frequent Q-value exchanges, the signaling overhead $O_{signaling}$ of the proposed method is reduced by an order of magnitude. Its normalized overhead is given by $O_{overhead}\approx \frac{T\times J}{T_{cycle}}$, where the overhead growth rate decelerates as the traffic demand increases.

Substituting Equation (1), i.e., $g_{k}=r_{k}-c_{k}$, into the objective function, and noting that $c_{k}$ includes the bandwidth $b_{k}$ and energy $e_{k}$, the greedy algorithm updates the remaining traffic demand R in each round (from the second round onward, as shown in line 26. This implies that $r_{k}$ decreases linearly with the number of times cell *K* is served, exhibiting diminishing marginal returns. Consequently, the algorithm is guaranteed to converge to a local optimum within the finite time horizon *T*. Moreover, owing to the convexity of the diminishing-return function, the total gain curve generated by this greedy strategy grows logarithmically and eventually flattens.

## 6. Graph Reinforcement Learning-Based Scheduling

### 6.1. Graph-Based Modeling

As presented above, the beam-hopping scheduling problem can be formulated as solving the maximum weighted independent set (MWIS) on a conflict graph at each time slot. However, because the MWIS is NP-hard and the LEO network topology evolves rapidly over time, conventional heuristic algorithms are generally unable to find the global optimum. To overcome this difficulty, this section introduces a graph reinforcement learning (GRL) framework that models the scheduling problem as a structured Markov Decision Process (MDP).

**Definition of a graph:** In the graph reinforcement learning (GRL)-based beam-hopping scheduling framework, the *K* cells within the satellite coverage area are modeled as a dynamic graph $G_{t}=\left(V,E_{t}\right)$, where

- $V=\{v_{1},v_{2},\ldots ,v_{k}\}$ denotes the set of nodes, with each node $v_{k}$ corresponding to a cell;
- $E_{t}=E_{t}^{int}\cup E_{t}^{aux}$ is the edge set, which consists of both interference edges and auxiliary edges.

The graph structure is dynamically updated at each scheduling time slot *t* to reflect variations in satellite positions, changes in beam coverage relationships, and fluctuations in traffic distribution.

The feature vector $f_{k}\left(t\right)$ of each cell node $v_{k}$ at time slot *t* is designed as:

(30) $$\begin{array}{cc} f_{k}\left(t\right)=[ & R_{k}\left(t\right),\rho_{k}^{hp}\left(t\right),Q_{k}\left(t\right),\tau_{k}\left(t\right),\\ & h_{k}\left(t\right),\Delta t_{k},P_{k},x_{t-1,k}]\in R^{8} \end{array}$$

All features are Z-score-normalized prior to inputting them to the graph neural network (GNN) to remove scale differences.

**Definition of interference edge $E_{t}^{\mathrm{int}}$:** Connect cell pairs that cannot be illuminated concurrently within the same time slot *t*, which is defined as

(31) $$\begin{array}{c} E_{t}^{int}=\left\{\left(m,n\right)|\theta_{mn}\left(t\right)<\theta_{c}\right\} \end{array}$$

Here, $\theta_{mn}\left(t\right)$ denotes the beam angle between cells *m* and *n* at time slot *t*, as computed by Equation (20), and $\theta_{c}$ is the co-channel interference threshold. When the angular separation between two cells, as seen from the satellite, is smaller than $\theta_{c}$, simultaneous illumination would incur unacceptable co-channel interference. Consequently, such cell pairs are subject to a conflict constraint, which is represented by edges in the graph. Owing to the high-speed motion of the satellite, $\theta_{mn}\left(t\right)$ varies over time, and thus the interference edge set $E_{t}^{int}$ is time-varying.

**Definition of auxiliary edge $E_{t}^{\mathrm{aux}}$:** Auxiliary edges are incorporated into the graph structure in addition to interference edges and are intended to augment information propagation.

To enhance the information propagation capability of the graph, three types of auxiliary edges are introduced as follows:

- Traffic association edges $E_{t}^{traffic}=\left\{\left(m,n\right)|corr\left(R_{m}\left(t\right),R_{n}\left(t\right)\right)\right\}$ connect cell pairs whose traffic demands exhibit strong temporal correlations (e.g., adjacent cities or cells within the same service area), thereby enabling the GNN to capture the spatiotemporal synergies in traffic patterns.
- Handover association edges $E_{t}^{handover}=\left\{\left(m,n\right)|p_{m\to n}<\eta_{ho}\right\}$ connect cell pairs that may exhibit user handover relationships. In LEO satellite networks, users frequently undergo handovers between adjacent cells due to satellite passage. Such auxiliary edges facilitate the model in learning the continuity of handover patterns.
- Spatial proximity edges $E_{t}^{geo}=\left\{\left(m,n\right)|d_{mn}<d_{th}\right\}$ is constructed based on the geographic distances $d_{mn}$ between cells, so as to ensure the connectivity of the graph structure, avoid isolated nodes, and facilitate the propagation of spatial local information.

### 6.2. Dynamic Graph Update

In the LEO satellite beam-hopping scheduling scenario, the graph structure is not static but evolves dynamically with satellite motion, channel variations, and traffic distribution. To this end, a comprehensive dynamic graph update mechanism must be designed to ensure that the graph neural network (GNN) obtains an accurate structured state representation at each time slot. The following provides a detailed exposition from five aspects: update trigger conditions, node feature updates, edge relationship updates, update strategy, and complexity analysis.

#### 6.2.1. Conditions for Triggering Graph Updates

The graph $G_{t}=\left(V,E_{t},F_{t}\right)$ is updated at each scheduling time slot *t*. The update triggering conditions are classified into two categories: mandatory updates and conditional updates.

- **Mandatory updates** are triggered at every time slot. The time-dependent components of the node features, such as the traffic demand $R_{k}\left(t\right)$, queue length $Q_{k}\left(t\right)$, and service interval $\tau_{k}\left(t\right)$, must be updated at each slot to reflect the real-time network state.
- **Conditional updates** are triggered only when specific conditions are met. The interference edge set $E_{t}^{int}$ is recomputed only when the satellite position change exceeds a threshold; the auxiliary edge set $E_{t}^{aux}$ is updated when the variation in traffic correlation or handover probability surpasses a threshold, see Table 1.

#### 6.2.2. Node Feature Updates

Among the components of the node feature matrix $F_{t}\in R^{K\times d}$, where *d* is the feature dimension of each node. According to the graph construction defined in the preceding section, the feature vector of each node $v_{k}$ consists of 8 components; therefore, d=8. The update frequencies and manners differ across distinct components, which is presented in the above table.

The queue length $Q_{k}\left(t\right)$ is updated according to the following equation,

(32) $$Q_{k}\left(t+1\right)=max\left\{0,Q_{k}\left(t\right)+A_{k}\left(t\right)-\mu_{k}\left(t\right)\cdot x_{k}\left(t\right)\right\}$$

where $A_{k}\left(t\right)$ is the volume of new data arrivals to cell *k* in time slot *t*, $\mu_{k}\left(t\right)$ is the service rate provided by a single beam to cell *k* at time slot *t* and $x_{k}\left(t\right)\in \left\{0,1\right\}$ denote the service indicator, with 1 indicating that cell *k* is served at time slot *t*, and 0 otherwise.

The update equation for the service interval $\tau_{k}\left(t\right)$ is given as follows,

(33) $$\tau_{k}\left(t+1\right)=\left\{\begin{array}{cc} 0, & x_{k}\left(t\right)=1\\ \tau_{k}\left(t\right)+1, & x_{k}\left(t\right)=0 \end{array}\right.$$

This update guarantees that the condition $\tau_{k}\left(t\right)\leq \Delta t_{k}$ specified in Constraint (7) is monitored in real time.

The channel gain $h_{k}\left(t\right)$ is updated as follows,

(34) $$h_{k}\left(t\right)=\frac{1}{N}\sum_{n=1}^{N}{\left|H_{k,n}\left(t\right)\right|}^{2}$$

where $H_{k,n}\left(t\right)$ denotes the downlink channel coefficient of the *n*th user in cell *k* during time slot *t*, which comprehensively accounts for path loss, shadow fading, and multipath effects. The downlink channel coefficient $H_{k,n}\left(t\right)$ in Equation (34) is modeled as the product of large-scale fading and small-scale fading. The detailed channel model and simulation parameters are given in Section 7.1.

#### 6.2.3. Edge Relationship Updates

Update of the interference edge set $E_{t}^{int}$: The interference edge set depends on the satellite position and is therefore time-varying. At each time slot *t*, the beam angles between all cell pairs must be recalculated according to the current satellite ephemeris,

(35) $$\theta_{mn}\left(t\right)=arccos\left(\frac{p_{sat}\left(t\right)\cdot p_{m}}{∥p_{sat}\left(t\right)∥\cdot ∥p_{m}∥}\cdot \frac{p_{sat}\left(t\right)\cdot p_{n}}{∥p_{sat}\left(t\right)∥\cdot ∥p_{n}∥}\right)$$

where $p_{sat}\left(t\right)$ is the satellite position vector, and $p_{m}$ and $p_{n}$ are the position vectors of the cell centers.

The update rule for the interference edges is as follows:

(36) $$e_{mn}^{int}\left(t\right)\in E_{t}^{int}⟺\theta_{mn}\left(t\right)<\theta_{c}$$

If the satellite position change between adjacent time slots is sufficiently small, i.e., $∥p_{sat}\left(t\right)-p_{sat}\left(t-1\right)∥<\epsilon$, then the interference edge set remains unchanged so as to reduce computational overhead.

Update of the auxiliary edge set $E_{t}^{aux}$: Auxiliary edges are classified into two categories, static and dynamic. The geographic proximity edges in $E_{t}^{geo}$ are static auxiliary edges, which are precomputed based on the fixed cell positions and are not updated. The traffic association edges $E_{t}^{traffic}$ are dynamic auxiliary edges, which are recomputed every $T_{corr}$ time slots based on sliding-window correlation. The handover association edges $E_{t}^{handover}$ are also dynamic auxiliary edges, which are updated every $T_{ho}$ time slot based on the user mobility model.

The traffic association edges are updated based on the Pearson correlation coefficient as Equation (33):

(37) $$corr_{mn}\left(t\right)=\frac{\sum_{s=t-W}^{t}\left(R_{m}\left(s\right)-{\bar{R}}_{m}\right)\left(R_{n}\left(s\right)-{\bar{R}}_{n}\right)}{\sqrt{\sum_{s=t-W}^{t}{\left(R_{m}\left(s\right)-{\bar{R}}_{m}\right)}^{2}}\sqrt{\sum_{s=t-W}^{t}{\left(R_{n}\left(s\right)-{\bar{R}}_{n}\right)}^{2}}}$$

If $corr_{mn}\left(t\right)>\eta_{corr}$, a traffic association edge is established between cells *m* and *n*.

In our paper, a soft update strategy is adopted, in which the magnitudes of variations in node features and edge relationships are examined at each time slot, and graph reconfiguration is triggered only when the change exceeds a preset threshold.

(38) $$\Delta F=∥F_{t}-F_{t-1}{∥}_{F}>\epsilon_{F}$$

(39) $$\Delta E=|E_{t}^{int}\setminus E_{t-1}^{int}\left|+\right|E_{t-1}^{int}\setminus E_{t}^{int}|>\epsilon_{E}$$

where $\epsilon_{F}$ denotes the preset threshold for triggering node feature updates, and $\epsilon_{E}$ denotes the threshold for triggering updates in response to changes in edge relationships.

When any of the conditions is satisfied, the graph structure is updated; otherwise, the graph structure from the previous time slot is inherited, and only the time-dependent components of the node features are updated.

The computational overhead of dynamic graph updates primarily arises from the recomputation of edges. By adopting the soft update strategy, the frequency of interference edge recomputation is reduced from every time slot to only when the satellite position change exceeds a threshold, thereby lowering the average complexity to $O(K^{2}/\alpha )$, where α denotes the time-interval ratio for position changes that exceed the threshold. For LEO satellites (with a typical visibility duration of about 5∼10 min), α is generally on the order of 5∼10, which substantially reduces the computational burden.

### 6.3. Graph Adjacency Matrix and Message Passing

In the graph $G_{t}=\left(V,E_{t}\right)$, the adjacency matrix $A_{t}\in R^{K\times K}$ is a numerical representation of the graph topology, whose entries are defined as follows,

(40) $$A_{t}\left[m,n\right]=\left\{\begin{array}{cc} 1, & \left(m,n\right)\in E_{t}^{int}\cup E_{t}^{aux}\;\mathrm{and}\;m\neq n\\ 1, & m=n\\ 0, & others \end{array}\right.$$

A self-loop is added to each node in the graph to ensure that the node’s own feature information is not diluted or lost during the message-passing process.

The adjacency matrix is formed by combining the following three sub-matrices,

(41) $$A_{t}=I+A_{t}^{int}+A_{t}^{aux}$$

where I is the identity matrix (corresponding to self-loops), $A_{t}^{int}$ is the interference adjacency matrix, with $A_{t}^{int}\left[m,n\right]=1$ if and only if $\theta_{mn}\left(t\right)<\theta_{c}$, $A_{t}^{aux}$ is the auxiliary adjacency matrix, with $A_{t}^{aux}\left[m,n\right]=1$ if and only if there exists a traffic association, handover association, or geographic proximity relationship.

Taking the simulation scenario with K=37 cells, where cells 11 and 31 are designated as high-priority cells, as an example, assume that the adjacency matrix structure at a given time slot is as follows:

(42) $$A_{t}={\left[\begin{array}{cccccc} 1 & 1 & 0 & \cdots & 0 & 0\\ 1 & 1 & 0 & \cdots & 0 & 0\\ 0 & 0 & 1 & \cdots & 0 & 1\\ \vdots & \vdots & \vdots & \ddots & \vdots & \vdots \\ 0 & 0 & 0 & \cdots & 1 & 1\\ 0 & 0 & 1 & \cdots & 1 & 1 \end{array}\right]}_{37\times 37}$$

Row 1: Cell 1 has a self-loop and an interference edge with cell 2. Row 3: Cell 3 has a self-loop and an auxiliary edge with cell 37. The matrix is symmetric (the graph is undirected).

Graph neural networks propagate information and update features on the graph structure through the message-passing mechanism. Each layer of message passing consists of three core steps as follows,

**Step 1: Message computation**

Each node $v_{k}$ collects information from its neighboring nodes u∈N(k) and generates a message,

(43) $$m_{u\to k}^{\left(l\right)}={\mathrm{MSG}}^{\left(l\right)}\left(h_{k}^{\left(l\right)},h_{u}^{\left(l\right)},e_{ku}\right)$$

where $h_{k}^{\left(l\right)}$ denotes the feature vector of node $v_{k}$ at the *l*th layer; $h_{u}^{\left(l\right)}$ is the feature vector of the neighbor node $v_{u}$; $e_{ku}$ is the edge feature of edge (k,u) (optional); and MSG is a learnable message function.

The edge feature $e_{ku}$ can be used to encode the type of edge (interference or auxiliary), though it is not mandatory; information may also be propagated purely via the graph topology.

**Step 2: Feature aggregation**

Each node collects the messages from all its neighboring nodes and aggregates them into a consolidated representation,

(44) $$a_{k}^{\left(l\right)}={\mathrm{AGG}}^{\left(l\right)}\left(\{m_{u\to k}^{\left(l\right)}:u\in N\left(k\right)\}\right)$$

The aggregation function AGG can take one of the following forms:

- Sum aggregation:
  (45) $$a_{k}^{\left(l\right)}=\sum_{u\in N(k)}m_{u\to k}^{\left(l\right)}$$
- Mean aggregation:
  (46) $$a_{k}^{\left(l\right)}=\frac{1}{\left|N(k)\right|}\sum_{u\in N(k)}m_{u\to k}^{\left(l\right)}$$
- Max aggregation:
  (47) $$a_{k}^{\left(l\right)}=\max_{u\in N(k)}m_{u\to k}^{\left(l\right)}$$
- Attention-based aggregation: a weighted sum, where the weights are learned via attention.

**Step 3: Node feature update**

The aggregated information is combined with the node’s own features to update the node representation,

(48) $$h_{k}^{\left(l+1\right)}={\mathrm{UPDATE}}^{\left(l\right)}\left(h_{k}^{\left(l\right)},a_{k}^{\left(l\right)}\right)$$

The update function commonly takes one of the following forms,

- A linear transformation with a non-linear activation:
  (49) $$h_{k}^{\left(l+1\right)}=\sigma \left(W^{\left(l\right)}a_{k}^{\left(l\right)}+b^{\left(l\right)}\right)$$
- Using a gated architecture such as a GRU:
  (50) $$h_{k}^{\left(l+1\right)}=\mathrm{GRU}\left(h_{k}^{\left(l\right)},a_{k}^{\left(l\right)}\right)$$

In this paper, we adopt the Graph Attention Network (GAT) as the graph encoder, whose core idea is to introduce learnable attention weights, enabling each node to adaptively attend to more important neighbors when aggregating information from its neighbors.

**Step 1: Attention coefficient computation**

At the *l*th layer of the GAT, the attention coefficient $\alpha_{ku}^{\left(l\right)}$ of node $v_{k}$ to its neighbor node $v_{u}$ is computed as Equation (47), where || denotes the vector concatenation operation; $a^{\left(l\right)}$ is the learnable attention parameter vector; $W^{\left(l\right)}$ is the learnable weight matrix; and LeakyReLU is the nonlinear activation function (with the negative slope typically set to 0.2).

(51) $$\alpha_{ku}^{\left(l\right)}=\frac{exp\left(\mathrm{LeakyReLU}\left(a^{(l)T}\left[W^{\left(l\right)}h_{k}^{\left(l\right)}\left|\right|W^{\left(l\right)}h_{u}^{\left(l\right)}\right]\right)\right)}{\sum_{v\in N(k)\cup k}exp\left(\mathrm{LeakyReLU}\left(a^{(l)T}\left[W^{\left(l\right)}h_{k}^{\left(l\right)}\left|\right|W^{\left(l\right)}h_{v}^{\left(l\right)}\right]\right)\right)}$$

**Step 2: Message aggregation and node update**

Based on the attention coefficients, node $v_{k}$ aggregates information from its neighbors via a weighted sum,

(52) $$h_{k}^{\left(l+1\right)}=\sigma \left(\sum_{u\in N(k)\cup \{k\}}\alpha_{ku}^{\left(l\right)}W^{\left(l\right)}h_{u}^{\left(l\right)}\right)$$

Multi-head attention is employed to improve the expressive power,

(53) $$h_{k}^{\left(l+1\right)}=|{|}_{r=1}^{H}\sigma \left(\sum_{u\in N(k)\cup \{k\}}\alpha_{ku}^{\left(l,r\right)}W^{\left(l,r\right)}h_{u}^{\left(l\right)}\right)$$

where *H* is the number of attention heads.

**Step 3: Multi-layer message passing and node embedding**

After *L* layers of GAT message passing, the final embedding $h_{k}^{\left(L\right)}$ of each node aggregates the structural information and node features within its *L*-hop neighborhood. To obtain a global representation of the entire graph (which serves as the state input to the policy network), a pooling operation is performed over all node embeddings,

(54) $$h_{G}^{\left(L\right)}=\mathrm{POOL}\left(\left\{h_{1}^{\left(L\right)},h_{2}^{\left(L\right)},\cdots ,h_{K}^{\left(L\right)}\right\}\right)$$

The pooling operations can take the following forms,

- Mean pooling:
  (55) $$h_{G}^{\left(L\right)}=\frac{1}{K}\sum_{k=1}^{K}h_{k}^{\left(L\right)}$$
- Max pooling:
  (56) $$h_{G}^{\left(L\right)}=\max_{k}h_{k}^{\left(L\right)}$$
- Attention pooling: a learnable weighted combination.

### 6.4. The Design of Algorithm

#### 6.4.1. Definitions of State, Action, and Reward

The state spaceis defined as the state observed by the agent at time slot *t*,

(57) $$s_{t}=\left\{F_{t},A_{t-1},Q_{t},\tau_{t},p_{t}^{sat}\right\}$$

where $F_{t}\in R^{K\times d}$ denotes the node embedding matrix extracted by the graph neural network, $A_{t-1}$ is the scheduling decision of the previous time slot (a *K*-dimensional binary vector), $Q_{t}$ represents the queue lengths of all cells, $\tau_{t}$ is the time elapsed since the last service for each cell, and $p_{t}^{sat}$ denotes the satellite position/ephemeris information.

Action Space: The action $a_{t}\in {\left\{0,1\right\}}^{K}$ is a *K*-dimensional binary vector indicating which cells are illuminated in the current time slot. It must satisfy the following constraints:

- The number of activated cells satisfies $\sum_{k}a_{t,k}\leq J$ (available beam constraint);
- The angular separation between any two activated cells satisfies $\theta_{mn}\geq \theta_{c}$ (interference constraint (5)).

Reward Function: The reward design should be consistent with the objective function in Equation (2),

(58) $$r_{t}=\sum_{k=1}^{K}a_{t,k}\cdot g_{k}\left(t\right)-\lambda_{1}\cdot c_{ht}-\lambda_{2}\cdot c_{bs}$$

where $g_{k}\left(t\right)=r_{k}\left(t\right)-c_{k}\left(t\right)$ is the system gain as defined in Definition 1; $c_{ht}$ is the penalty for high-priority service timeout, i.e., violation of Constraint (7); $c_{bs}$ denotes the cost of beam-switching overhead.

#### 6.4.2. Graph Neural Network Architecture

We adopt the Graph Attention Network (GAT) as the encoder to map the raw node features into structured embeddings,

(59) $$h_{v}^{l+1}=\sigma \left(\sum_{u\in N(v)}\alpha_{vu}^{\left(l\right)}W^{\left(l\right)}h_{u}^{\left(l\right)}\right)$$

The attention coefficient $\alpha_{vu}$ is computed as,

(60) $$\alpha_{vu}=\frac{exp\left(\mathrm{LeakyReLU}\left(a^{T}[Wh_{v}||Wh_{u}]\right)\right)}{\sum_{w\in N(v)}exp\left(\mathrm{LeakyReLU}\left(a^{T}[Wh_{v}||Wh_{w}]\right)\right)}$$

The final node embeddings $h_{v}^{\left(L\right)}$ are pooled to obtain the graph-level representation $h_{G}$.

#### 6.4.3. Graph Reinforcement Learning Algorithm

We propose a graph reinforcement learning (GRL) framework to address the beam-hopping scheduling problem in low-Earth-orbit (LEO) satellite networks. The scheduling problem is formulated as a structured Markov decision process (MDP) on a dynamic graph, where each cell corresponds to a node, and edges represent interference constraints (interference edges) as well as auxiliary relationships (traffic, handover, and spatial proximity) that facilitate information propagation.

At each time slot, the agent observes a state composed of node embeddings extracted by a Graph Attention Network (GAT) encoder, previous scheduling decisions, queue lengths, service intervals, and satellite ephemerides. The GAT computes attention coefficients to adaptively aggregate neighborhood information, and multi-layer message passing yields node embeddings that are pooled into a graph-level representation. The action is a binary vector indicating which cells are illuminated, subject to beam and interference constraints. The reward function combines system gains, a severe penalty for high-priority service timeouts, and a switching overhead penalty, thereby aligning with the original optimization objective. The policy network is trained via reinforcement learning to maximize cumulative rewards, enabling adaptive and near-optimal scheduling decisions under time-varying topologies and traffic conditions, see Algorithm 2.
**Algorithm 2** Graph reinforcement learn-based scheduling algorithm (GRL-BHS).**Require:** Graph neural network parameters $\theta_{GNN}$, policy network parameters $\theta_{\pi }$, value network parameters $\theta_{Q}$, experience replay buffer D.  1:**for** each scheduling episode $episode=1,\cdots ,N_{ep}$ **do**  2:    The initial graph state $G_{1}$ is obtained, and the queue $Q_{1}$ is initialized.  3:    **for** each time slot t=1,⋯,I **do**  4:      Extract node embeddings using the GAT encoder: $H_{t}={\mathrm{GAT}}_{\theta_{GNN}}\left(G_{t}\right)$.  5:      Generate a scheduling decision via the policy network: $a_{t}∼\pi_{\theta_{\pi }}\left(\cdot |H_{t},s_{t}\right)$, and      apply the masking mechanism to ensure that constraints (4)∼(7) are satisfied.  6:      Execute action $a_{t}$, update the queue and system state, and compute the immediate      reward $r_{t}$.  7:      Store the experience $\left(s_{t},a_{t},r_{t},s_{t+1}\right)$ into D.  8:      **if** |D|≥batch_size **then**  9:         Sample a mini-batch of experiences from D.10:         Update the parameters of the GNN, policy network, and value network using         Proximal Policy Optimization (PPO) or Soft Actor–Critic (SAC).11:      **end if**12:    **end for**13:**end for**

#### 6.4.4. Constraint Handling Mechanism

To ensure that the scheduling decisions satisfy all the hard constraints proposed in this paper, an action masking strategy is adopted.

**Masking unavailable beams:** If beam *j* has already been assigned to another cell at time slot *t*, the corresponding action $a_{jk}\left(t\right)$ is masked.

**Masking high-priority timeout cells:** If cell *k* has reached $\Delta t_{k}$ time slots since its last service, it is forced into the candidate set (corresponding to Constraint (7)).

**Masking interference-conflicting cells:** If the angular separation between cell *k* and any already activated cell in the current time slot is less than $\theta_{c}$, cell *k* is removed from the action space (corresponding to Constraint (5)).

**Masking cells that have reached the maximum allocation limit:** If cell *k* has already been allocated P(k)·T time slots, no further allocation is made (corresponding to Constraint (3)).

**Masking cells that have not reached the minimum allocation:** When the number of remaining slots is only sufficient to satisfy the minimum allocation $\tau^{\prime }$ of the unserved cells, those cells are forced into the candidate set and other low-priority cells are masked (Constraint (6)).

**Masking unprofitable cells:** If $g_{k}\left(t\right)=r_{k}\left(t\right)-c_{k}\left(t\right)<=0$, cell *k* is removed from the action space (Constraint (10)).

This masking mechanism ensures that the action $a_{t}$ output by the agent is always physically feasible, without requiring any post-processing correction.

#### 6.4.5. Training Hyperparameters and Reproducibility

To ensure reproducibility, the key hyperparameters of the GRL-BHS training process are summarized in Table 2. We adopted Proximal Policy Optimization (PPO) as the policy optimization algorithm, since the action space was discrete and PPO provides stable training with a clipped surrogate objective. The Graph Attention Network (GAT) encoder used 2 layers with 4 attention heads and a hidden dimension of 64. The policy and value networks were both implemented as 2-layer fully connected networks with 128 hidden units and ReLU activations.

The training was performed offline for 500 episodes. Each episode consisted of T=20 time slots. The experience replay buffer size was 10,000, and the mini-batch size was 64. The learning rate was set to $3\times 10^{-4}$ for both the actor and critic networks, and the discount factor was γ=0.99. The GAE parameter was λ=0.95, and the PPO clip range was ϵ=0.2. The entropy coefficient was 0.01 to encourage exploration. The target network was updated every 100 gradient steps. All experiments were conducted on a workstation with an NVIDIA RTX-3090 GPU and an Intel Core i9-12900K CPU.

These hyperparameters were selected based on a grid search over the learning rate $\left\{10^{-4},\;3\times 10^{-4},\;10^{-3}\right\}$, batch size {32,64,128}, and GAT layers {1,2,3}. The selected configuration achieved the best trade-off between convergence speed and final reward. The source code and trained model will be made available upon reasonable request, see Table 2.

### 6.5. Theoretical Analysis of Performance

#### 6.5.1. Stability Condition

We adopt the Lyapunov optimization framework and define a virtual queue as

(61) $$Z_{k}\left(t+1\right)=\max\left\{Z_{k}\left(t\right)+R_{k}^{r}\left(t\right)-\mu_{k}\left(t\right),0\right\}$$

where $\mu_{k}\left(t\right)$ is the service rate. If $\rho_{k}=E\left[R_{k}^{r}\right]/E\left[\mu_{k}\right]<1$, the system is stable in the mean sense, and the queue lengths are bounded.

#### 6.5.2. Convergence Analysis

Under standard reinforcement learning assumptions (learning rates satisfying the Robbins–Monro conditions, a finite action space, and bounded rewards), the policy evaluation and policy improvement steps of the GRL-BHS algorithm converge in probability to the optimal action-value function and the optimal policy, respectively.

#### 6.5.3. Complexity Analysis

**Forward inference complexity:** The GAT encoder incurs O(L·|E|·d) complexity, while the policy network requires O(K·d). The overall complexity is therefore O(T·|E|·d+T·K·d).

**Comparison with HBSA:** The online inference complexity of GRL is approximately O(K·d), which is significantly lower than the O(T×J×K) complexity of HBSA. The training phase is performed offline, but once trained, the model can be rapidly deployed.

#### 6.5.4. Approximate Performance Bound

Owing to the ability of graph neural networks to capture global structural information, the GRL policy can theoretically achieve a (1−1/e) approximation ratio to the MWIS solution (under the submodularity condition), which outperforms the 1/(Δ+1) bound of greedy algorithms as given in Equation (25).

#### 6.5.5. Constraint Handling Framework

The optimization problem in (2)–(10) is a single-objective constrained problem. Instead of relaxing all constraints into the objective via penalty terms, we adopt a feasibility-preserving framework: hard constraints are enforced during the construction of the scheduling decision, while soft operational penalties are incorporated only into the reward of GRL-BHS. The handling of each constraint is summarized as follows.

Constraint (3) (fairness): In HBSA, each cell is assigned a weight proportion $P_{k}$, and the number of slots allocated to cell *k* is bounded by $P_{k}\cdot T$. In GRL-BHS, once cell *k* has been allocated $P_{k}\cdot T$ slots, its action is masked.

Constraint (4) (available beams): HBSA checks whether beam *j* is idle at slot *t* and ensures that at most one cell is illuminated by one beam. GRL-BHS masks any beam already assigned in the current slot, so that $\sum_{k}a_{t,k}<=J$.

Constraint (5) (interference): HBSA calculates the angle $\theta_{mn}\left(t\right)$ between candidate beams and rejects any assignment with $\theta_{mn}\left(t\right)<\theta_{c}$. GRL-BHS masks cells whose angular separation from any already activated cell is below $\theta_{c}$.

Constraint (6) (minimum slot allocation): In HBSA, each cell is initialized with a minimum reservation $\tau^{\prime }$. The greedy selection first serves cells whose minimum reservation has not been met. In GRL-BHS, cells that have not reached $\tau^{\prime }$ are forced into the candidate set when the remaining slots are tight.

Constraint (7) (delay guarantee for high-priority services): HBSA pre-schedules high-priority cells according to their maximum tolerable interval $\Delta t_{k}$, as shown in lines 4–16 of Algorithm 1. GRL-BHS masks high-priority cells that have reached $\Delta t_{k}$ since their last service, forcing them to be scheduled.

Constraint (8) (binary variable): $x_{jk}\left(t\right)$ is defined as a binary decision variable, and both HBSA and GRL-BHS generate only 0/1 actions.

Constraint (9) (non-negative cost): $c_{k}>=0$ is guaranteed by the cost definition.

Constraint (10) (gain not less than cost): In HBSA, a candidate cell is scheduled only if $w_{k}>=c_{k}$; otherwise it is removed from the temporary candidate set. In GRL-BHS, cells with $g_{k}\left(t\right)<=0$ are masked.

For HBSA, all hard constraints are checked before each assignment, so the output H is always feasible. For GRL-BHS, the action masking mechanism in Section 6.4.4 ensures that the policy output is physically feasible without post-processing.

## 7. Simulations

### 7.1. Simulation Settings and Example

Within a given hopping beam cycle, there exists a set of time slots. A total of six full-band beams were configured, with Beam 1 to Beam 6 corresponding to red, yellow, orange, green, blue and purple respectively. With a 1 GHz spectrum bandwidth, one time slot of the hopping beam could satisfy one unit of service demand. The cell configuration is illustrated in Figure 2a, and the corresponding service demand of each cell is presented in Figure 2b. The service weight set is expressed as R= {12, 38, 1, 17, 15, 30, 31, 7, 19, 17, 25, 28, 30, 11, 27, 26, 6, 4, 19, 0, 13, 5, 8, 30, 10, 20, 27, 35, 0, 21, 22, 8, 10, 33, 10, 32, 9}. The corresponding weight proportion set is P= {0.0183, 0.0579, 0.0015, 0.0259, 0.0229, 0.0457, 0.0473, 0.0107, 0.0290, 0.0259, 0.0381, 0.0427, 0.0457, 0.0168, 0.0412, 0.0396, 0.0091, 0.0061, 0.0290, 0, 0.0198, 0.0076, 0.0122, 0.0457, 0.0152, 0.0305, 0.0412, 0.0534, 0, 0.0320, 0.0335, 0.0122, 0.0152, 0.0503, 0.0152, 0.0488, 0.0137}. For non-adjacent illuminated cells, the included angle between corresponding beams is consistently greater than the interference threshold. Cell 11 and 31 bear high-priority and time-sensitive service requirements, which are marked with shaded areas in Figure 2b. Specifically, the maximum beam-hopping interval of cell 11 is limited to one, while the maximum beam-hopping interval of cell 31 is restricted to zero. The system gain obtained by activating each cell is positively proportional to its service weight, and the system gain set is assumed as to be $G_{s}=$ {1.2, 3.8, 0.1, 1.7, 1.5, 3.0, 3.1, 0.7, 1.9, 1.7, 2.5, 2.8, 3.0, 1.1, 2.7, 2.6, 0.6, 0.4, 1.9, 1.2, 3.8, 0.1, 1.7, 1.5, 3.0, 3.1, 0.7, 1.9, 1.7, 2.5, 2.8, 3.0, 1.1, 2.7, 2.6, 0.6, 0.4, 1.9}.

The downlink channel coefficient $H_{k,n}\left(t\right)$ in Equation (34) is modeled as the product of large-scale fading and small-scale fading,

(62) $$H_{k,n}\left(t\right)=\sqrt{L_{k,n}\left(t\right)}\cdot g_{k,n}\left(t\right)$$

where $L_{k,n}\left(t\right)$ denotes the large-scale channel gain, and $g_{k,n}\left(t\right)$ denotes the small-scale fading coefficient. The large-scale gain in dB is expressed as,

(63) $$L_{k,n}^{dB}\left(t\right)=20log_{10}\left(\frac{4\pi d_{k,n}\left(t\right)f_{c}}{c}\right)+X_{\sigma }+A_{rain}\left(\theta_{k,n}\left(t\right)\right)$$

where $d_{k,n}\left(t\right)$ is the slant distance between the satellite and the *n*th user in cell *k*, $f_{c}$ is the carrier frequency, *c* is the speed of light, $X_{\sigma }∼N\left(0,\sigma^{2}\right)$ is the shadowing term with standard deviation σ, and $A_{rain}\left(\theta \right)$ is the rain attenuation depending on the elevation angle θ. The path-loss exponent was set to α=2, corresponding to free-space propagation in LEO satellite links. The small-scale fading $g_{k,n}\left(t\right)$ followed a Rician distribution with K-factor *K*, which captures the dominant line-of-sight component and the multipath effects. Then, $h_{k}\left(t\right)$ in Equation (34) is the average channel power gain over all users in cell *k*.

In the simulations, the carrier frequency was set to $f_{c}=20$ GHz (Ka-band), the shadowing standard deviation was σ=3 dB, the Rician K-factor was K=10 dB, and the elevation angle ranged from 30° to 90°. Rain attenuation was modeled according to the ITU-R P. 618 model. These settings are typical for LEO satellite downlink transmissions and are consistent with the channel gain definition in Equation (34).

**Interference threshold:** The co-channel interference angle threshold was set to $\theta_{c}=30{}^{\circ}$, which is approximately 1.5 times the 3 dB beamwidth of the satellite antenna. Two cells can be illuminated simultaneously only if the angular separation between their beams is at least $\theta_{c}$.

**Traffic model:** User traffic was generated according to a non-uniform spatial distribution, where hotspot cells had higher demand than rural cells. The traffic arrival process was modeled as a Poisson process with time-varying arrival rate. High-priority and time-sensitive services accounted for 20% and 10% of the total traffic, respectively. Bursty traffic was generated by a Markov-modulated Poisson process (MMPP) to emulate temporary events such as competitions and disasters.

**Load definition:** The normalized traffic load is defined as

(64) $$\rho =\frac{\sum_{k\in K}R_{k}}{T\times J\times R_{b}}$$

where $R_{k}$ is the total traffic demand of cell *k*, *T* is the number of slots per scheduling cycle, *J* is the number of beams, and $R_{b}$ is the maximum service rate of one beam per slot. In the simulations, ρ ranged from 0.4 to 1.2, corresponding to light to heavy load.

**Statistical validation:** Each simulation was repeated 100 times with independent random seeds. The results are reported as the mean value with 95% confidence intervals. The paired t-test and Wilcoxon signed-rank test were used to verify whether the performance differences between HBSA and the baselines were statistically significant (p<0.05), see Table 3.

Upon receiving all input conditions, the beam-hopping pattern can be calculated via Algorithm 1. Figure 3 illustrates the results of the first three rounds of beam-hopping scheduling. Each beam (Beam 1 to Beam 6) is represented by a distinct pattern for clear identification. Assuming 20 beam-hopping time slots within each scheduling cycle, the six beams collectively occupy 120 time slots in total.

In the first round of scheduling, Beams 1 and 2 are first deployed to serve cells 11 and 31, respectively, which are assigned with high priority and carry time-sensitive traffic. The remaining cells are then scheduled by adopting a heuristic scheduling algorithm. Starting from cell 2, which yields the maximum weighted gain, Beams 3, 4, 5, and 6 are sequentially assigned to serve cells 2, 26, 34, and 23, while taking into account factors such as inter-beam co-channel interference and fairness.

After updating the weighted gains of all cells, the second round of scheduling is initiated. Since cell 11 can be re-served after an interval of one time slot, Beam 1 is preferentially allocated to serve cell 31. The remaining cells are served by Beams 2 to 6 based on their updated weighted gains.

Subsequently, the third round of scheduling commences. As both cells 11 and 31 require priority service in this round, Beam 1 is first dispatched to serve cell 11, followed by Beam 2 for cell 31, according to their respective weighted gains. The remaining cells are then covered by Beams 3 to 6 using the aforementioned heuristic algorithm.

In view of the practical application scenarios of the beam-hopping system for LEO constellations, this paper adopted the aforementioned beam-hopping scheduling scheme oriented to high-priority and time-sensitive services. On this basis, beams were scheduled preferentially to guarantee the transmission requirements of high-priority and time-sensitive services. Meanwhile, by conducting benefit–cost accounting, the system gain of the beam-hopping system was formally defined. Furthermore, a novel beam-hopping scheduling algorithm based on system gain was proposed in LEO satellite networks. The proposed method enables beam scheduling that better conforms to actual engineering operation conditions.

### 7.2. Simulation Results

With the experimental scenario and parameter settings depicted in Figure 2, simulation experiments were carried out to analyze the system gain and user satisfaction, with a particular focus on the QoS-guarantee performance of high-priority and time-sensitive services. It should be noted that the same hard constraint $▵t_{k}$ applied to the HBSA was also imposed on the Round-Robin and MWC schemes, ensuring a fair basis for performance comparison.

Figure 4 depicts the system gain versus the total service demand for the seven considered scheduling schemes. As the service demand increases from 200 to 2000 units, all algorithms exhibit a monotonic increase in system gain, yet with markedly different growth rates and saturation levels. The proposed HBSA consistently outperforms the Round-Robin, EDF, ACO, and GA schemes across the entire demand range. Notably, HBSA achieves a system gain of 10.8 at low demand (200 units), which is approximately twice that of Round-Robin (5.4) and EDF (6.0), and it maintains a stable lead of about 35∼40% over these baseline schemes as demand grows. When compared with the near-optimal MWC algorithm, HBSA achieves comparable performance at moderate to high loads (e.g., 15.2 vs. 15.2 at 1200, and 16.8 vs. 16.8 at 2000), while incurring significantly lower computational complexity. The GRL-BHS scheme, leveraging graph reinforcement learning, provides the highest system gain across all load levels (from 10.5 at 200 to 17.0 at 2000), slightly surpassing both MWC and HBSA. However, the performance gap between GRL-BHS and HBSA remains within 2% for most load conditions, confirming that HBSA achieves near-optimal gain with much lower implementation overhead. These results validate the effectiveness of the proposed HBSA in delivering high system gain while maintaining practical feasibility for LEO satellite networks.

Figure 5 illustrates the user satisfaction level across 37 cells for the seven considered scheduling schemes. As shown in the table, the vast majority of cells achieve 100% satisfaction under all algorithms, indicating that each scheme is capable of meeting user demands in most cells under the given simulation settings. However, notable exceptions are observed in cell 1, where the Round-Robin scheme yields a satisfaction of only 52%, while all other algorithms (EDF, ACO, GA, HBSA, MWC, and GRL-BHS) achieve between 80% and 84%. This significant discrepancy suggests that Round-Robin, lacking any priority or load-awareness mechanism, fails to allocate sufficient resources to this particular cell, which presumably has a disproportionately high traffic demand or unfavorable channel conditions. In contrast, the proposed HBSA achieves 80% satisfaction in cell 1, comparable to MWC and GRL-BHS, confirming its ability to adapt to non-uniform traffic distributions. Another interesting observation is cell 29, where GA attains 128% satisfaction, which may indicate an overservice scenario where the algorithm allocates more resources than strictly required, reflecting the less efficient resource utilization of evolutionary approaches. Overall, Figure 4 demonstrates that HBSA consistently delivers satisfaction levels on par with the near-optimal MWC and GRL-BHS schemes across all cells, while substantially outperforming Round-Robin in challenging cells, thereby validating its effectiveness in ensuring user-level quality of service.

Figure 6 depicts the system overhead versus the total service demand for the seven considered scheduling schemes. The overhead values are presented on a logarithmic scale, with all algorithms exhibiting a general increasing trend as the demand grows from 200 to 2000 units. However, the growth rates differ considerably among the schemes. The proposed HBSA consistently maintains the lowest overhead across the entire demand range, with values around $1.0\times 10^{1}$ across all load levels, demonstrating its stable and efficient resource utilization. In contrast, the MWC algorithm, while achieving near-optimal system gain, incurs substantially higher overhead, with its overhead increasing from $1.0\times 10^{1}$ at low demand to over $3.0\times 10^{1}$ at high demand (as indicated by the parenthetical values), reflecting the exponential complexity of its clique-search-based optimization. The Round-Robin and EDF schemes maintain relatively constant overhead, yet their overhead levels are consistently higher than that of HBSA, due to inefficient beam utilization and increased signaling interactions. The GRL-BHS scheme, leveraging graph reinforcement learning, achieves performance comparable to HBSA in terms of overhead (around $1.0\times 10^{1}$ for most load conditions), confirming its practical feasibility for onboard deployment, provided that the inference engine is appropriately optimized. Overall, Figure 5 demonstrates that HBSA strikes the most favorable balance between system performance and operational overhead, particularly under heavy-load conditions, where its overhead growth rate is significantly slower than that of MWC and other benchmark algorithms, thereby confirming its practical deployability in resource-constrained LEO satellite platforms.

In addition, the proposed scheme achieves a favorable balance of time slot allocation between low-gain and high-gain regions, thereby generating greater system benefits. Furthermore, it boosts the overall system gain and enhances the satisfaction of high-priority and time-sensitive users. Meanwhile, the system overhead is reduced, and an optimal comprehensive performance balance of the entire system is realized.

To quantify the fairness of resource allocation among cells for different algorithms, we adopt the Jain’s fairness index, defined as,

(65) $$J=\frac{{\left(\sum_{k=1}^{K}x_{k}\right)}^{2}}{K\sum_{k=1}^{K}x_{k}^{2}}$$

where $x_{k}$ denotes the number of time slots allocated to cell *k* during the scheduling period. The index ranges from 1/K to one, with a value closer to unity indicating a more equitable distribution of resources.

Figure 7 presents the Jain fairness index versus the normalized traffic load for the seven considered scheduling schemes. As the traffic load increases from 0.4 to 1.2, all algorithms exhibit a declining trend in fairness, yet with considerably different rates of decrease. The Round-Robin scheme maintains an almost perfect fairness index of approximately 0.99 across all load levels, as it allocates resources in a strictly Round-Robin manner regardless of traffic variations. The EDF scheme follows closely, with a gradual decline from 0.99 to 0.91, reflecting its deadline-aware prioritization which still preserves reasonable fairness. The proposed HBSA achieves a fair index of 0.92 at low load, decreasing to 0.84 at high load, which represents a well-balanced trade-off between system gain and fairness. The GRL-BHS scheme exhibits a slightly lower fairness index than HBSA, ranging from 0.90 to 0.82, because its reinforcement learning policy tends to favor high-gain cells more aggressively in pursuit of higher system gain. The GA and ACO schemes show moderate fairness degradation, from 0.88 and 0.84 down to 0.78 and 0.76, respectively, due to their inherent stochastic search biases. The MWC algorithm, which exclusively maximizes total weighted gain, yields the lowest fairness across all load levels (from 0.76 to 0.68), confirming that its greedy preference for high-weight cells inevitably sacrifices equity among cells. Overall, Figure 6 demonstrates that HBSA strikes a favorable balance between fairness and system gain, achieving competitive fairness comparable to GRL-BHS and GA, while substantially outperforming MWC, thereby validating its effectiveness in ensuring equitable resource allocation across cells with non-uniform traffic demands.

To evaluate the convergence behavior of the algorithms used in this study, we plot the convergence curves of GRL-BHS, GA, ACO and HBSA in Figure 8, with a unified horizontal axis from zero to 200 iterations. Since the four algorithms optimize different performance metrics, the vertical axis is normalized to the final value of each curve, so that all curves lie in the range [0, 1] and can be compared directly. As shown in Figure 8, GRL-BHS converges after about 150 episodes, GA converges after about 80 generations, and ACO converges after about 120 iterations. HBSA is a deterministic greedy construction algorithm; its cumulative system gain saturates after the first 20 time slots and remains constant thereafter. For visual comparison, its curve is extended to 200 iterations. MWC is an exact clique-search benchmark, while Round-Robin and EDF are deterministic rules; therefore, no iterative convergence curves are provided for these three schemes.

### 7.3. Scalability to Multi-Satellite Scenarios

The simulations in Section 7.2 considered a single satellite with 37 cells to validate the core scheduling logic of HBSA. In practical mega-constellations, however, hundreds of beams may be distributed across multiple satellites. This subsection discusses how HBSA scales to such multi-satellite scenarios.

Complexity scaling: For a constellation with *S* satellites, let satellite *s* have $J_{s}$ beams and $K_{s}$ cells. If each satellite runs HBSA independently, the total complexity is

(66) $$O\left(\sum_{s=1}^{S}T\times J_{s}\times K_{s}\right)=O\left(T\times J_{total}\times K_{avg}\right)$$

which grows linearly with the number of satellites and remains polynomial. Since $J_{s}$ and $K_{s}$ are bounded by the on-board antenna and coverage configuration, the per-satellite complexity is unchanged. Even for S=100 satellites with $J_{s}=10$ beams and $K_{s}=50$ cells, the total complexity is still far below the exponential complexity of MWC.

Inter-satellite coordination overhead: HBSA adopts a centralized pre-computation approach. The hopping pattern of each satellite is computed at the ground station or a network operation control center (NOCC), and only the scheduling triplets t,j,k need to be broadcast. The signaling overhead per satellite is $O(T\times J_{s}\times log_{2}K_{s})$ bits per scheduling cycle. For S satellites, the total overhead is $O(S\times T\times J\times log_{2}K)$ bits, which is transmitted once at the beginning of each cycle. In contrast, online DRL schemes require frequent Q-value exchanges among satellites, resulting in an order-of-magnitude higher signaling overhead. Therefore, HBSA remains attractive for multi-satellite deployment.

Inter-satellite interference coordination: In multi-satellite scenarios, co-channel interference may arise between beams of different satellites. This can be handled by extending the conflict graph: each vertex represents a cell, and an interference edge is added between two cells if their angular separation from their respective satellites is below the threshold $\theta_{c}$. HBSA’s greedy maximum-weight-first strategy can still be applied to this extended graph. Since the maximum degree $\phi_{max}$ is bounded by the antenna beamwidth and the spatial isolation between satellites, the approximation bound $1/(\phi_{max}+1)$ remains valid. Alternatively, a hierarchical scheduling architecture can be adopted: the NOCC allocates frequency/time resources among satellites to avoid inter-satellite interference, while each satellite independently runs HBSA for its own cells. This decouples the problem and keeps the coordination overhead low.

## 8. Conclusions and Future Work

### 8.1. Conclusions

This paper addressed the beam-hopping scheduling problem in LEO satellite networks with a focus on high-priority and time-sensitive services by establishing a system-gain maximization model and proposing a heuristic beam scheduling algorithm (HBSA) that pre-schedules time-critical traffic and greedily allocates remaining resources under interference and fairness constraints, achieving a desirable trade-off between performance and complexity. Extensive simulations, benchmarked against Round-Robin, EDF, ACO, GA, MWC, and an extended graph-reinforcement-learning scheme (GRL-BHS), demonstrated that HBSA delivered near-optimal system gain (comparable to MWC and GRL-BHS with a gap of less than 2% under most load conditions), ensured 100% satisfaction for high-priority services, maintained a Jain fairness index above 0.84 even under heavy load, and reduced signaling overhead by an order of magnitude compared to online learning approaches, all while preserving a low polynomial complexity of O(T×J×K) that is practically deployable on resource-constrained LEO platforms. While the GRL-BHS extension offered the highest gain at the cost of offline training and moderate overhead, HBSA stood out as the most balanced and immediately viable solution for real-world LEO satellite systems, providing a solid foundation for future intelligent and adaptive beam-hopping scheduling in mega-constellations.

### 8.2. Future Work

Although the proposed heuristic algorithm shows promising performance, several aspects can be further explored to enhance beam-hopping scheduling in LEO mega-constellations.

**Adaptive Beam-Hopping Pattern Design:** The current model assumes a fixed number of beams and time slots. Future research can extend it to an adaptive framework that dynamically adjusts the beam-hopping pattern according to real-time traffic prediction and satellite mobility. This would enable the system to better match time-varying service demands and channel conditions, further improving resource utilization.

**Deep Reinforcement Learning for Distributed Scheduling:** The heuristic algorithm presented in this paper relies on centralized ground control. Future work can integrate deep reinforcement learning (DRL) techniques, allowing each satellite or even each beam to act as an agent that learns an optimal scheduling policy in a distributed manner. Such an approach would reduce signaling overhead, enhance scalability, and improve robustness against dynamic constellation topologies.

**Coordination with Non-Orthogonal Multiple Access and Full-Duplex Communication:** The synergy between beam-hopping and advanced transmission schemes, such as non-orthogonal multiple access (NOMA) and full-duplex communication, remains largely unexplored. Future studies can investigate joint optimization of beam-hopping patterns with NOMA clustering and power allocation, as well as full-duplex operation, to further boost spectral efficiency and reduce latency for high-priority services.

**Multi-Satellite Scalability Validation:** The current simulations focus on a single satellite with 37 cells. Future work will extend the simulation to multi-satellite constellations with hundreds of beams, including inter-satellite coordination overhead and cross-satellite interference, to further validate the scalability of HBSA.

**Federated Learning for Distributed Scheduling:** Federated learning (FL) is an emerging paradigm for distributed satellite scheduling, where each satellite or beam agent trains a local model on its own traffic and channel data and only exchanges model updates with the ground station or neighboring satellites [36]. This avoids raw data transmission, reduces signaling overhead, and preserves user privacy. Combined with the GRL-BHS framework, FL could enable scalable and privacy-aware beam hopping scheduling in mega-constellations, where each satellite learns a local policy and periodically aggregates model parameters to adapt to global traffic dynamics.

**Quantum Computing for Combinatorial Optimization:** The beam hopping scheduling problem is essentially a maximum weighted independent set (MWIS) problem, which is NP-hard. Quantum computing, particularly quantum annealing and the quantum approximate optimization algorithm (QAOA), offers a new paradigm for solving such combinatorial optimization problems [37]. In the future, quantum-assisted or quantum-inspired algorithms could accelerate the search for near-optimal beam-hopping patterns and further reduce the computational burden on resource-constrained LEO satellites. Although current quantum hardware is not yet mature for on-board deployment, quantum-inspired heuristics and hybrid quantum-classical methods are promising directions for ground-based scheduling centers.

**Impact of Inter-Satellite Links on Beam-Hopping Scheduling:** In mega-constellations, inter-satellite links (ISLs) enable data relaying among satellites and may significantly affect traffic flow and resource allocation. Future work should incorporate ISL constraints and opportunities into the beam hopping scheduling model, considering end-to-end delay, multi-hop routing, and load balancing across the whole network.

**Experimental Validation Using Hardware-in-the-Loop or Real Satellite Testbeds:** While simulation results demonstrate the effectiveness of the proposed method, practical fading, handover procedures, and signaling delays can affect real-world performance. Future efforts should focus on experimental validation using hardware-in-the-loop (HIL) platforms or actual satellite testbeds, thereby assessing the algorithm’s feasibility and robustness under realistic operational conditions.

## Figures and Tables

**Figure 1:**
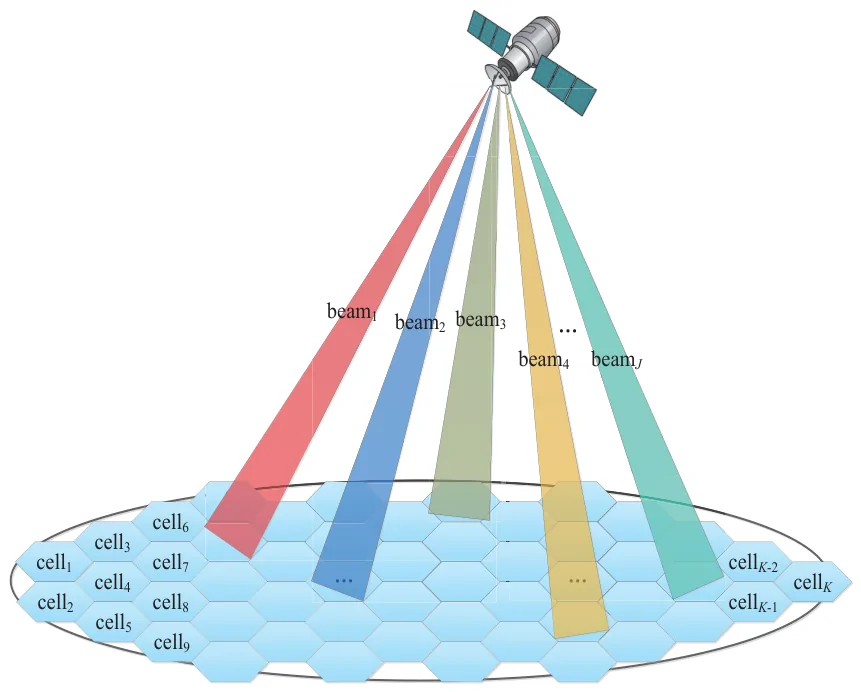
LEO beam-hopping satellite communication system.

**Figure 2:**
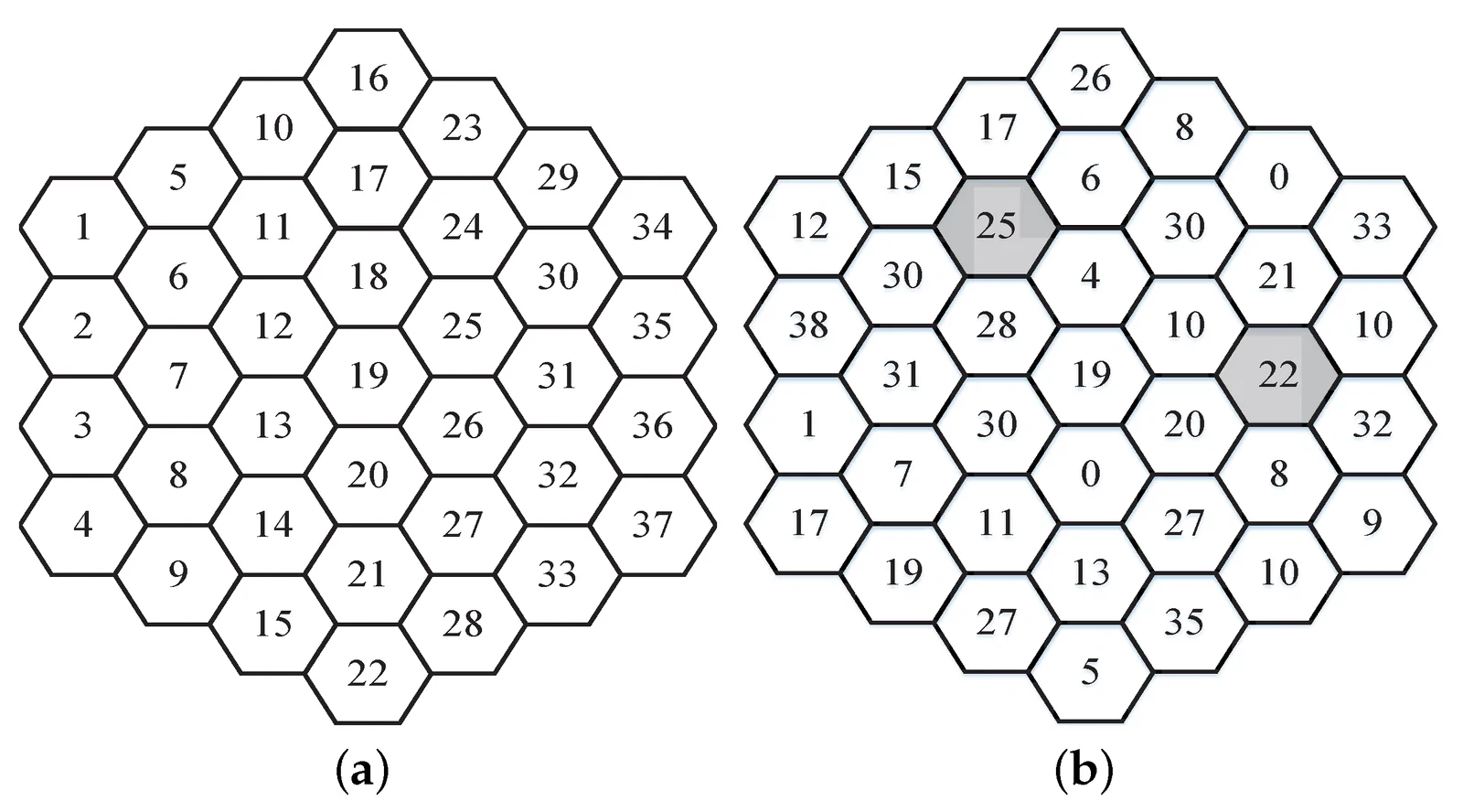
Configuration and initial service traffic of cells. (**a**) Configuration of cells. (**b**) Initial service traffic of cells.

**Figure 3:**
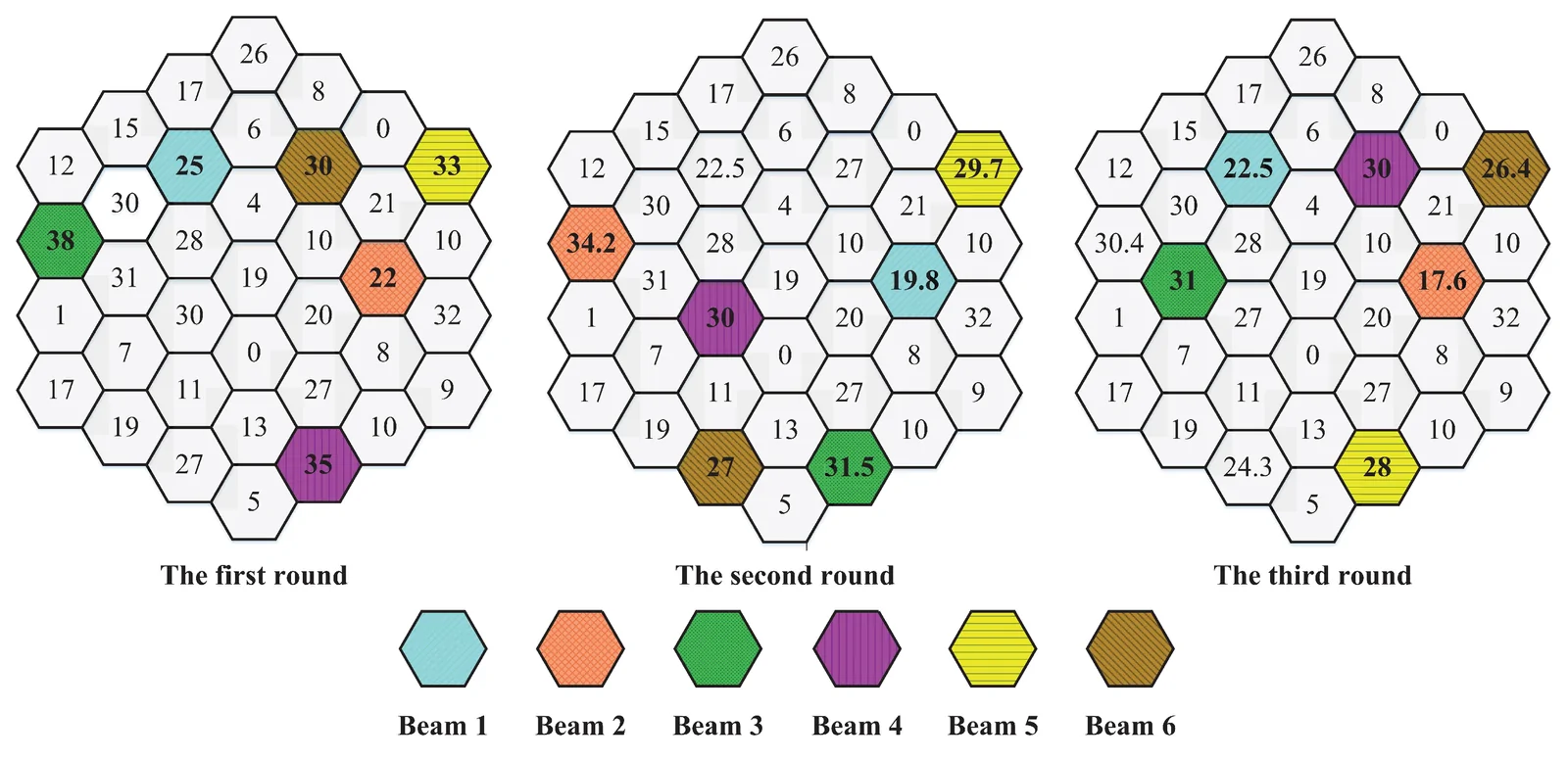
Example of beam-hopping scheduling.

**Figure 4:**
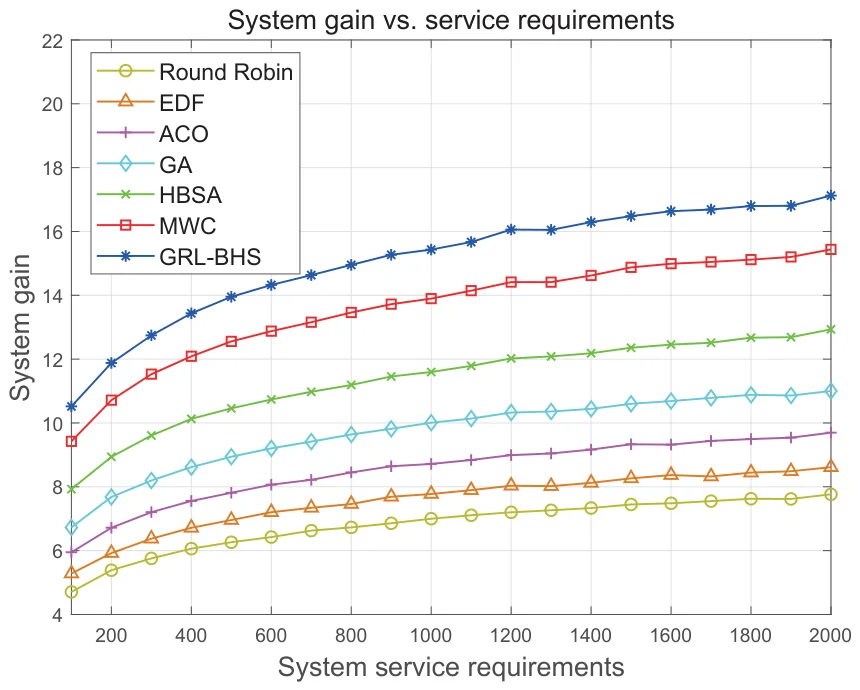
Simulation result of system gain vs. service requirements.

**Figure 5:**
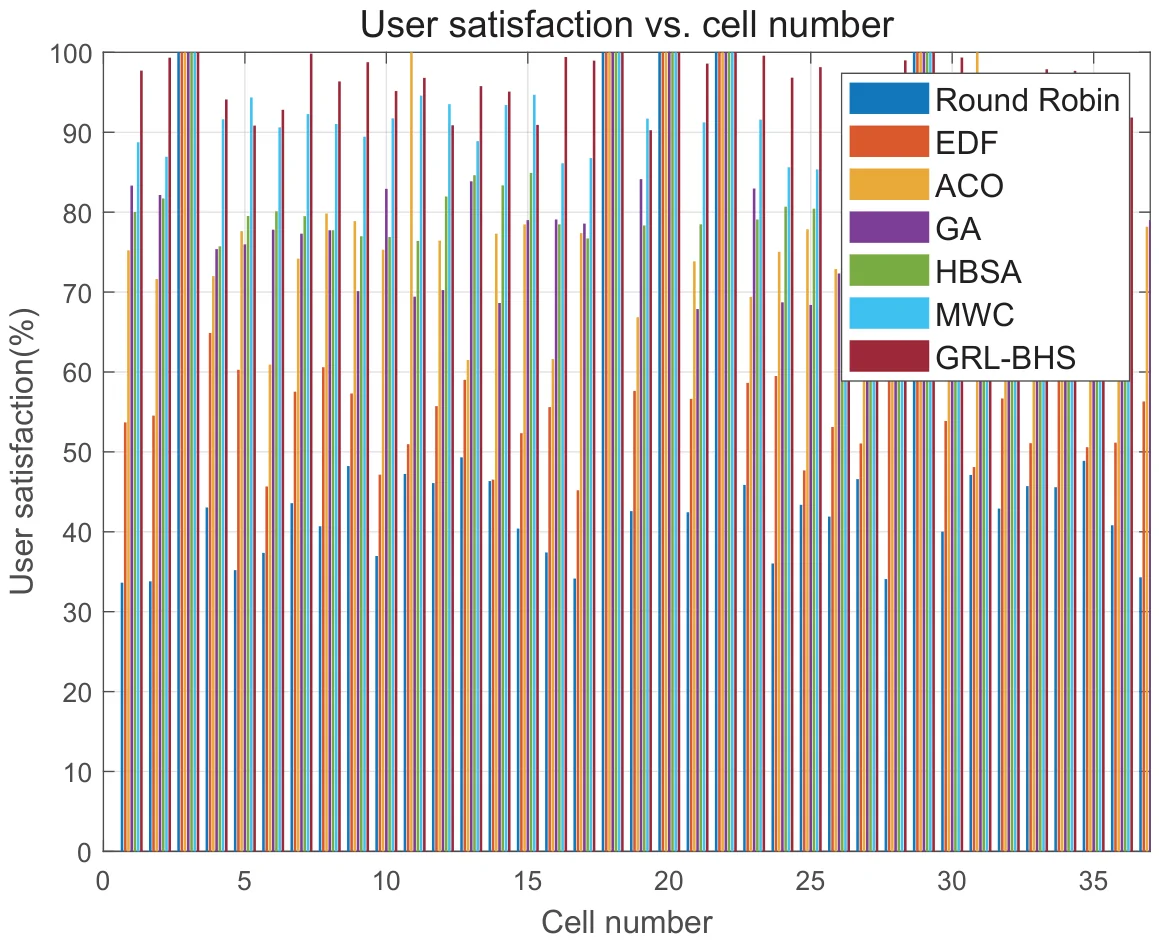
Simulaiton result of user satisfaction vs. cell number.

**Figure 6:**
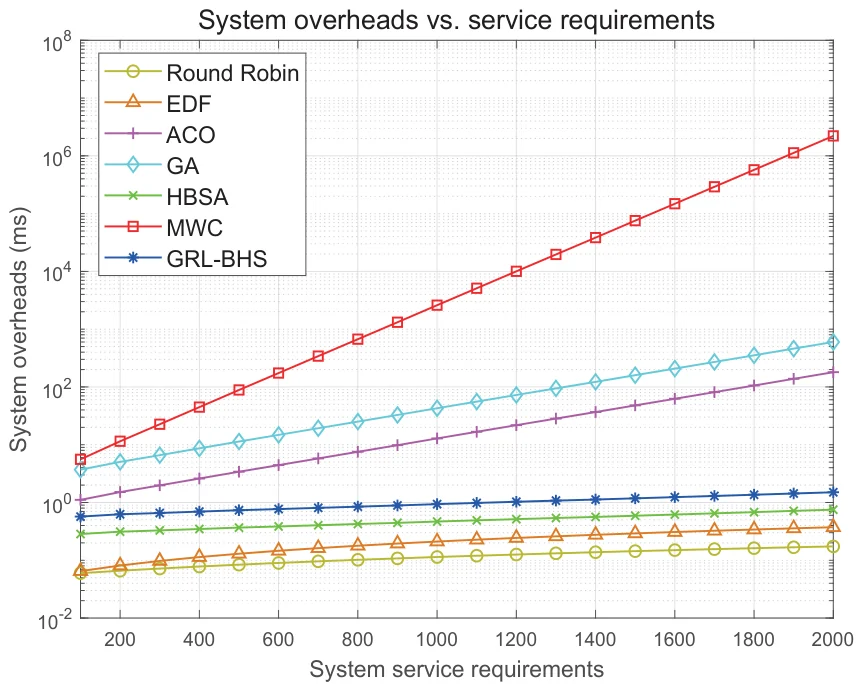
Simulation result of system overheads vs. service requirements.

**Figure 7:**
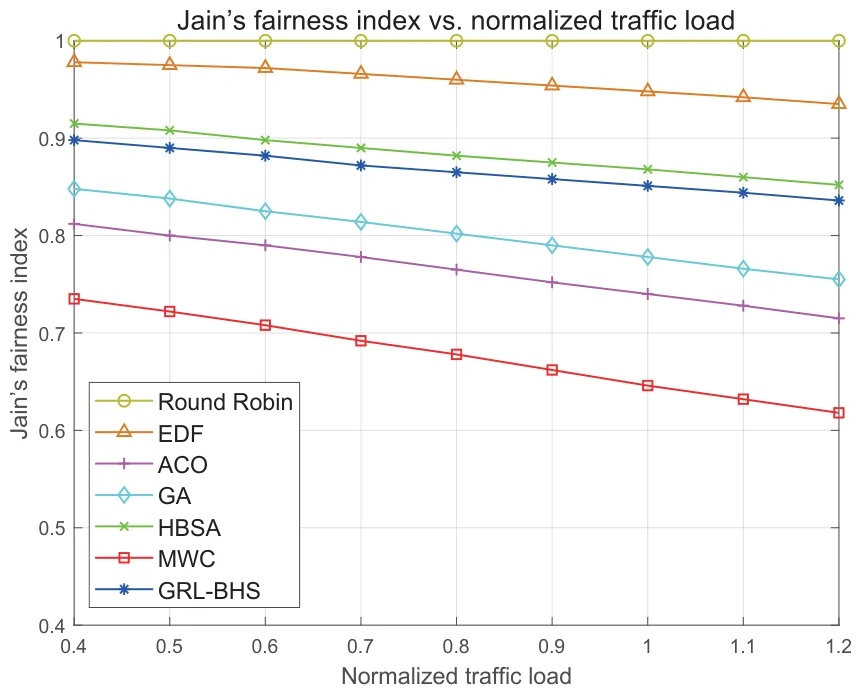
Simulation result of Jain’s fairness index vs. normalized traffic load.

**Figure 8:**
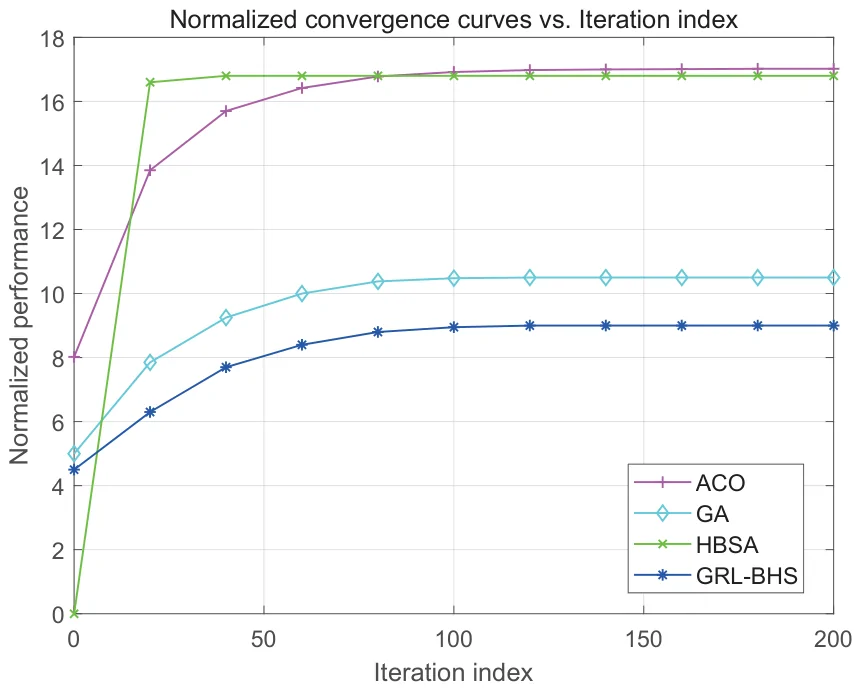
Normalized convergence curves of GRL-BHS, GA, ACO, and HBSA with a unified horizontal axis (0–200 iterations).

**Table 1:** Node feature updates.

Feature Components	Symbols	Update Approaches	Update Frequency
Traffic demand	$R_{k}\left(t\right)$	Acquired or predicted in real time from the ground gateway	Each time slot
Proportion of high-priority traffic	$\rho_{k}^{hp}\left(t\right)$	Calculated based on queue statistics	Each time slot
Buffer queue length	$Q_{k}\left(t\right)$	Updated dynamically based on the service state and arrival process	Each time slot
The service interval, i.e., the number of time slots elapsed since cell *k* was last served	$\tau_{k}\left(t\right)$	$\tau_{k}\left(t+1\right)=\tau_{k}\left(t\right)+1$ if not served; 0 if served	Each time slot
Channel gain	$h_{k}\left(t\right)$	Derived from satellite positions and propagation models	At each time slot or over a longer period
Maximum tolerable interval	$\Delta t_{k}$	Static parameter and is not updated over time	Constant
Weight for fairness	$P_{k}$	Static parameter and is not updated over time	Constant
Previous-slot service status	$x_{k}\left(t-1\right)$	$x_{k}\left(t-1\right)=x_{k}\left(t-2\right)$ (sliding update)	Each time slot

**Table 2:** Training hyperparameters of GRL-BHS.

Hyperparameter	Value
Reinforcement learning algorithm	PPO
Number of training episodes	500
Time slots per episode	20
GAT layers	2
GAT attention heads	4
GAT hidden dimension	64
Policy/value network hidden units	128 (2 layers)
Activation function	ReLU
Learning rate (actor and critic)	$3\times 10^{-4}$
Discount factor γ	0.99
GAE parameter λ	0.95
PPO clip range ϵ	0.2
Entropy coefficient	0.01
Replay buffer size	10,000
Mini-batch size	64
Target network update interval	100 gradient steps
Optimizer	100 Adam
Hardware	NVIDIA RTX-3090 GPU, Intel Core i9-12900K

**Table 3:** Simulation parameters.

Parameter	Value
Orbit altitude	550 km
Orbital inclination	53°
Carrier frequency	20 GHz (Ka-band)
Path-loss exponent α	2
Shadowing standard deviation σ	3 dB
Rician K-factor	10 dB
Rain attenuation model	ITU-R P.618
Elevation angle	30°∼90°
Interference threshold $\theta_{c}$	3°
High-priority traffic ratio	20%
Time-sensitive traffic ratio	10%
Traffic arrival	Poisson/MMPP
Number of runs	100
Confidence interval	95%

## Data Availability

The original contributions presented in this study are included in the article. Further inquiries can be directed to the corresponding author.
